# Supplementary material for: Understanding indirect assortative mating and its intergenerational consequences for educational attainment
Source: Nat Commun. 2025 Jun 6;16:5264. doi: 10.1038/s41467-025-60483-0 (PMC12144155; doi:10.1038/s41467-025-60483-0)
Supplement: Supplementary file 2 — Reporting Summary [file 41467_2025_60483_MOESM2_ESM.pdf]

nature portfolio

Corresponding author(s): Hans Fredrik Sunde

Last updated by author(s): May 9, 2025

Reporting Summary

Nature Portfolio wishes to improve the reproducibility of the work that we publish. This form provides structure for consistency and transparency in reporting. For further information on Nature Portfolio policies, see our Editorial Policies and the Editorial Policy Checklist.

Statistics

For all statistical analyses, confirm that the following items are present in the figure legend, table legend, main text, or Methods section.

n/a	Confirmed	
☐	☒	The exact sample size (*n*) for each experimental group/condition, given as a discrete number and unit of measurement
☐	☒	A statement on whether measurements were taken from distinct samples or whether the same sample was measured repeatedly
☐	☒	The statistical test(s) used AND whether they are one- or two-sided<br>*Only common tests should be described solely by name; describe more complex techniques in the Methods section.*
☐	☒	A description of all covariates tested
☐	☒	A description of any assumptions or corrections, such as tests of normality and adjustment for multiple comparisons
☐	☒	A full description of the statistical parameters including central tendency (e.g. means) or other basic estimates (e.g. regression coefficient) AND variation (e.g. standard deviation) or associated estimates of uncertainty (e.g. confidence intervals)
☐	☒	For null hypothesis testing, the test statistic (e.g. *F*, *t*, *r*) with confidence intervals, effect sizes, degrees of freedom and *P* value noted<br>*Give P values as exact values whenever suitable.*
☒	☐	For Bayesian analysis, information on the choice of priors and Markov chain Monte Carlo settings
☐	☒	For hierarchical and complex designs, identification of the appropriate level for tests and full reporting of outcomes
☐	☒	Estimates of effect sizes (e.g. Cohen's *d*, Pearson's *r*), indicating how they were calculated

Our web collection on statistics for biologists contains articles on many of the points above.

Software and code

Policy information about availability of computer code

Data collection	Data was attained from population-wide administrative registers
Data analysis	Data was analysed in R 4.2.3. Models were estimated using OpenMx44 2.20.6. Scripts for running the models are available at https://osf.io/dznbk/

For manuscripts utilizing custom algorithms or software that are central to the research but not yet described in published literature, software must be made available to editors and reviewers. We strongly encourage code deposition in a community repository (e.g. GitHub). See the Nature Portfolio guidelines for submitting code & software for further information.

Data

Policy information about availability of data

All manuscripts must include a data availability statement. This statement should provide the following information, where applicable:

- Accession codes, unique identifiers, or web links for publicly available datasets
- A description of any restrictions on data availability
- For clinical datasets or third party data, please ensure that the statement adheres to our policy

The raw data are protected and are not available due to data privacy laws. The data for this study encompasses educational outcomes and demographic information for entire cohorts of the Norwegian population. Researchers can access the data by application to the Norwegian Regional Committees for Medical and

Health Research Ethics and the data owners (Statistics Norway and The Norwegian Institute of Public Health). The authors cannot share these data with other researchers.

Research involving human participants, their data, or biological material

Policy information about studies with human participants or human data. See also policy information about sex, gender (identity/presentation), and sexual orientation and race, ethnicity and racism.

Reporting on sex and gender	Sex or gender were not considered in this manuscript
Reporting on race, ethnicity, or other socially relevant groupings	Race, ethnicity, or other socially relevant groupings were not considered in this manuscript.
Population characteristics	Our sample comprise 212,070 x 2 Norwegian born families with opposite-sex parents and with children born between 1975 and 1995. Average birth year was M = 1956.0 (SD = 7.3) for fathers and M = 1958.7 (SD = 6.9) for mothers. Because we were interested in similarity between opposite-sex partners, both women and men are equally represented in the data.
Recruitment	Data on educational attainment and on family trees was attained from administrative registers that comprise the entire Norwegian population. As such, they are likely free from most forms of participation bias (selection into families notwithstanding). Zygosity on twins was attained from The Norwegian Twin Register, which is a consent-based register build of earlier twin studies. Details on recruitment is described elsewhere (Nilsen et. al., 2019. Twin Research and Human Genetics, 22(6), 647-650). The twin register may suffer from some selection bias.
Ethics oversight	The study was approved, and participant consent was waived by the (Norwegian) Regional Committee for Medical and Health Research Ethics (project #2018/ 434).

Note that full information on the approval of the study protocol must also be provided in the manuscript.

Field-specific reporting

Please select the one below that is the best fit for your research. If you are not sure, read the appropriate sections before making your selection.

☐ Life sciences ☒ Behavioural & social sciences ☐ Ecological, evolutionary & environmental sciences

For a reference copy of the document with all sections, see nature.com/documents/nr-reporting-summary-flat.pdf

Behavioural & social sciences study design

All studies must disclose on these points even when the disclosure is negative.

Study description	A cross-sectional population-wide quantitative family study
Research sample	The Norwegian population register, which comprise practically every citizen and inhabitant of Norway since 1960 until data release.
Sampling strategy	The data comprise the entire Norwegian population. Within these data, we investigated parents of children born between 1975 and 1995. We limited our analyses to Norwegian-born families with available educational data on both parents and children. For nuclear families with three or more children born in this time window, we randomly selected two children to include. All individuals that could be linked to extended families were included.
Data collection	Data on educational attainment was attained from administrative registers. Data on zygosity of twins was attained from the Norwegian Twin Register, who in turn have assessed it using questionnaires and, for a sub-sample, blood tests.
Timing	We included data on families with children born between 1975 and 1995. Had we extended the period to later births, the children would not yet be old enough to have attained education themselves. Had we extended the period to earlier births, the data on parents would become of lower quality.
Data exclusions	We investigated complete nuclear families with children born between 1975 and 1995. We limited our analyses to Norwegian-born families with available educational data on both parents and children. Individuals that did not meet these criteria were not included. Individuals who could not be linked to extended families were not included. If a nuclear family included more than two eligible children, we randomly selected two children and excluded the rest.
Non-participation	N/A
Randomization	We identified nuclear family units via shared parentage, randomly choosing two children for larger nuclear families. We then linked the nuclear families into extended family units via one of the parents' twin or sibling. We first linked together units by monozygotic and dizygotic twins and included twin uncles and aunts without eligible children themselves (to increase statistical power). For the remaining nuclear families, we linked them together via one of the parents' siblings, choosing randomly if there were multiple candidates. No individual formed part of more than one extended family unit.

Reporting for specific materials, systems and methods

We require information from authors about some types of materials, experimental systems and methods used in many studies. Here, indicate whether each material, system or method listed is relevant to your study. If you are not sure if a list item applies to your research, read the appropriate section before selecting a response.

Materials & experimental systems

n/a	Involved in the study
☒	☐ Antibodies
☒	☐ Eukaryotic cell lines
☒	☐ Palaeontology and archaeology
☒	☐ Animals and other organisms
☒	☐ Clinical data
☒	☐ Dual use research of concern
☒	☐ Plants

Methods

n/a	Involved in the study
☒	☐ ChIP-seq
☒	☐ Flow cytometry
☒	☐ MRI-based neuroimaging

Plants

Seed stocks	N/A
Novel plant genotypes	N/A
Authentication	N/A
