# Supplementary material for: Understanding indirect assortative mating and its intergenerational consequences for educational attainment
Source: Nat Commun. 2025 Jun 6;16:5264. doi: 10.1038/s41467-025-60483-0 (PMC12144155; doi:10.1038/s41467-025-60483-0)
Supplement: Supplementary file 4 — Source Data [file 41467_2025_60483_MOESM4_ESM.zip › Source Data/Fig1.pdf]

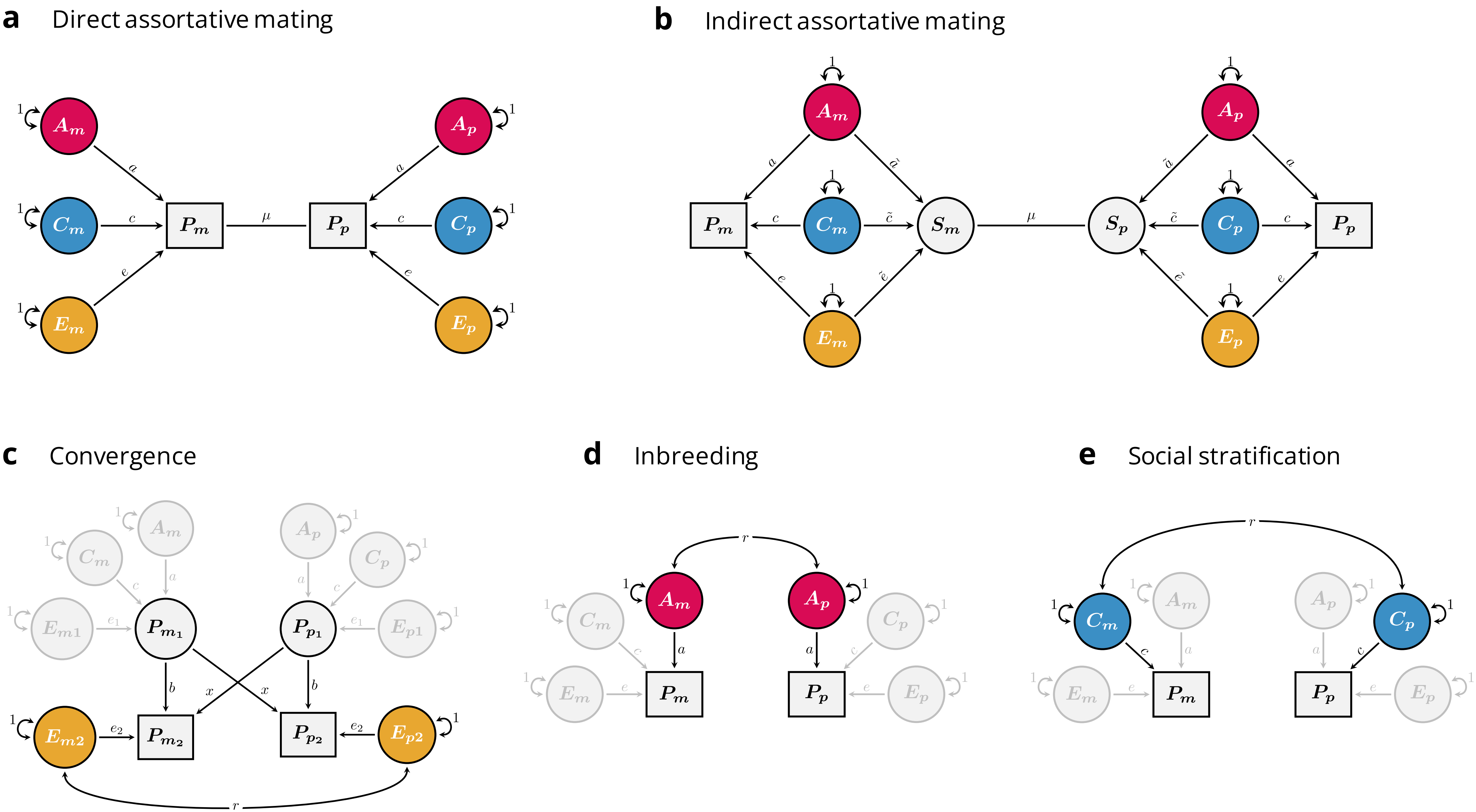
a
Direct assortative mating
A_m
C_m
E_m
P_m
μ
P_p
A_p
C_p
E_p
a
c
e
1
b
Indirect assortative mating
A_m
C_m
E_m
P_m
S_m
μ
S_p
A_p
C_p
E_p
P_p
$\tilde{a}$
$\tilde{c}$
$\tilde{e}$
c
Convergence
A_m
C_m
E_{m1}
P_{m_1}
A_p
C_p
E_{p1}
P_{p_1}
E_{m2}
P_{m_2}
P_{p_2}
E_{p2}
e_1
e_2
b
x
r
d
Inbreeding
A_m
A_p
C_m
C_p
E_m
E_p
P_m
P_p
r
e
Social stratification
C_m
A_m
A_p
C_p
E_m
E_p
P_m
P_p
r
