# Supplementary material for: Understanding indirect assortative mating and its intergenerational consequences for educational attainment
Source: Nat Commun. 2025 Jun 6;16:5264. doi: 10.1038/s41467-025-60483-0 (PMC12144155; doi:10.1038/s41467-025-60483-0)
Supplement: Supplementary file 4 — Source Data [file 41467_2025_60483_MOESM4_ESM.zip › Source Data/fig2.pdf]

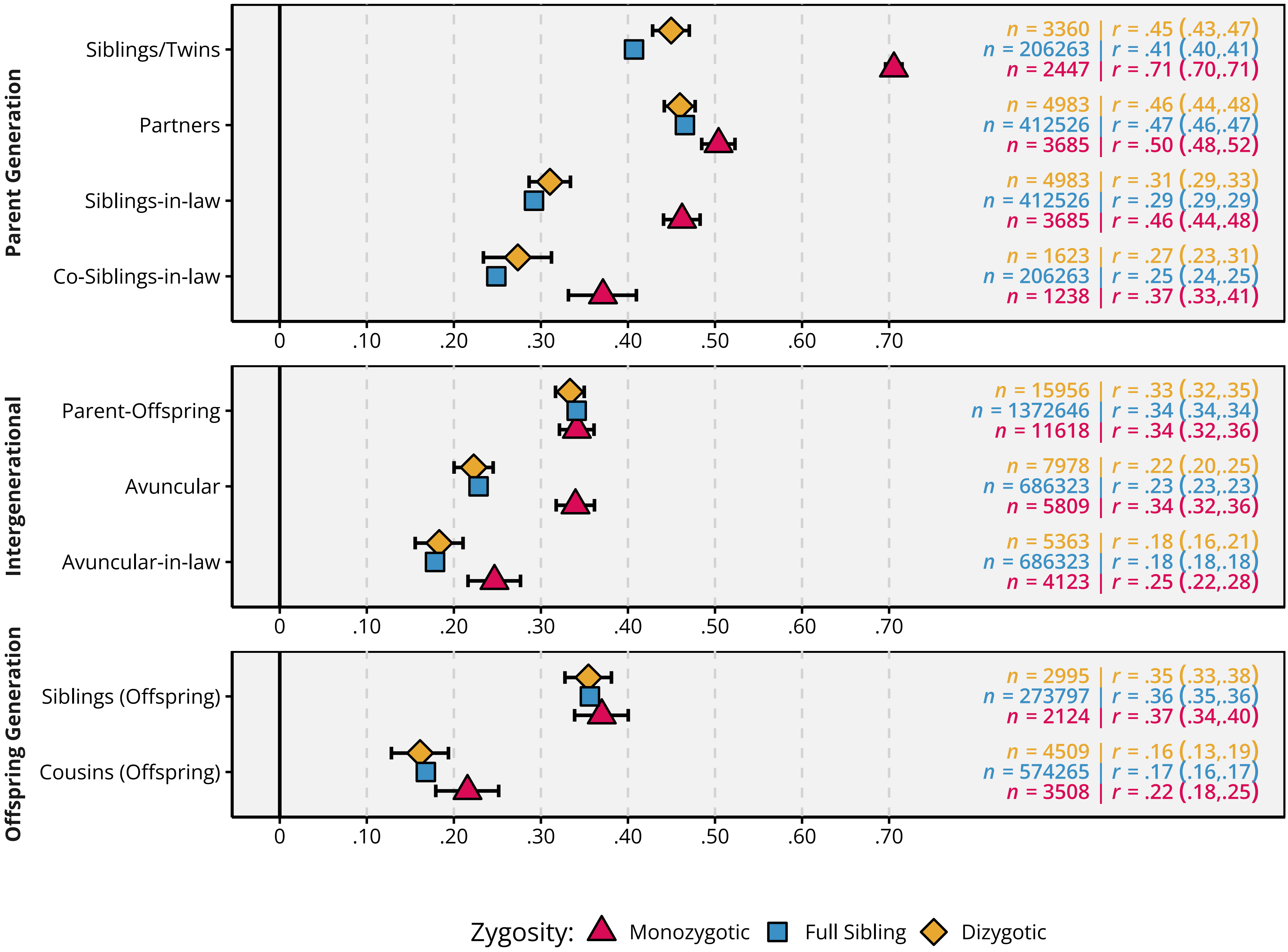
Parent Generation
Siblings/Twins
Partners
Siblings-in-law
Co-Siblings-in-law
n = 3360 | r = .45 (.43,.47)
n = 206263 | r = .41 (.40,.41)
n = 2447 | r = .71 (.70,.71)
n = 4983 | r = .46 (.44,.48)
n = 412526 | r = .47 (.46,.47)
n = 3685 | r = .50 (.48,.52)
n = 4983 | r = .31 (.29,.33)
n = 412526 | r = .29 (.29,.29)
n = 3685 | r = .46 (.44,.48)
n = 1623 | r = .27 (.23,.31)
n = 206263 | r = .25 (.24,.25)
n = 1238 | r = .37 (.33,.41)
0 .10 .20 .30 .40 .50 .60 .70
Intergenerational
Parent-Offspring
Avuncular
Avuncular-in-law
n = 15956 | r = .33 (.32,.35)
n = 1372646 | r = .34 (.34,.34)
n = 11618 | r = .34 (.32,.36)
n = 7978 | r = .22 (.20,.25)
n = 686323 | r = .23 (.23,.23)
n = 5809 | r = .34 (.32,.36)
n = 5363 | r = .18 (.16,.21)
n = 686323 | r = .18 (.18,.18)
n = 4123 | r = .25 (.22,.28)
0 .10 .20 .30 .40 .50 .60 .70
Offspring Generation
Siblings (Offspring)
Cousins (Offspring)
n = 2995 | r = .35 (.33,.38)
n = 273797 | r = .36 (.35,.36)
n = 2124 | r = .37 (.34,.40)
n = 4509 | r = .16 (.13,.19)
n = 574265 | r = .17 (.16,.17)
n = 3508 | r = .22 (.18,.25)
0 .10 .20 .30 .40 .50 .60 .70
Zygosity: Monozygotic Full Sibling Dizygotic
