# Supplementary material for: Understanding indirect assortative mating and its intergenerational consequences for educational attainment
Source: Nat Commun. 2025 Jun 6;16:5264. doi: 10.1038/s41467-025-60483-0 (PMC12144155; doi:10.1038/s41467-025-60483-0)
Supplement: Supplementary file 4 — Source Data [file 41467_2025_60483_MOESM4_ESM.zip › Source Data/iAM-COTS-equations.docx]

| **Equations implied by the iAM-COTS model in Figure S20** | | | |
| --- | --- | --- | --- |
| **Name** | **Shorthand** | **Equation** | **Note** |
| **Useful Shorthands** | | | |
| ***Variance Components in Parent generation*** | | | |
| Additive genetic variance | $V_{A1}=$ | $a_{1}^{2}$ |  |
| Sibling-shared environmental variance | $V_{C1}=$ | $c_{1}^{2}$ |  |
| Twin-shared environmental variance | $V_{T1}=$ | $t_{1}^{2}$ | $r_{t_{MZ}}=r_{t_{DZ}}=1;r_{t_{FS}}=0$ |
| Gene-Environment covariance | $V_{rGE1}=$ | $2\omega a_{1}c_{1}$ |  |
| Non-shared environmental variance | $V_{E1}=$ | $e_{1}^{2}$ |  |
| ***Variance Components in Offspring generation*** | | | |
| Additive genetic variance   associated with parental phenotype | $V_{A1P}=$ | $a'_{1}^{2}(f_{FS}+k)$ | $f_{FS}+k=1$ |
| Family variance   associated with parental phenotype | $V_{F}=$ | $2\sigma_{p}^{2}p^{2}+2c'_{1}^{2}+4p(c_{1}+a_{1}\omega)c'_{1}+2\mu(c'_{1}(c_{1}+a_{1}\omega)+\sigma_{PS}p)^{2}$ | *Both direct and passive environmental effects* |
| Additive genetic variance   independent of parental phenotype | $V_{A2}=$ | $a_{2}^{2}$ |  |
| Shared environmental variance   independent of parental phenotype | $V_{C2}=$ | $c_{2}^{2}$ |  |
| Gene-Environment covariance | $V_{rGE2}=$ | $2p(a_{1}+c_{1}\omega)a'_{1}+2a'_{1}\omega c'_{1}+2a'_{1}(\tilde{a}_{1}+\tilde{c}_{1}\omega)\mu((\tilde{c}_{1}+\tilde{a}\omega)c'_{1}+\sigma_{PS}p)$ |  |
| Unique environmental variance | $V_{E2}=$ | $e_{2}^{2}$ |  |
| ***Other useful Shorthands*** | | | |
| Correlation between P and S | $\sigma_{PS}=$ | $(\tilde{a}_{1}+\tilde{c}_{1}\omega)a_{1}+(\tilde{c}_{1}+\tilde{a}_{1}\omega)c_{1}+t_{1}\tilde{t}_{1}+e_{1}\tilde{e}_{1}$ | *Focal phenotype - Sorting factor correlation* |
| Sibling Correlation between P and S | $\delta_{PS}=$ | $(f\tilde{a}_{1}+\tilde{c}_{1}\omega)a_{1}+(\tilde{c}_{1}+\tilde{a}_{1}\omega)c_{1}+\tilde{t}_{1}r_{t}t_{1}$ | *Correlation between focal phenotype  and their siblings sorting factor* |
| Sibling Correlation between S and S | $\delta_{SS}=$ | $f\tilde{a}_{1}^{2}+\tilde{c}_{1}^{2}+2\tilde{a}_{1}\omega\tilde{c}_{1}+r_{t}\tilde{t}_{1}^{2}$ | *Correlation between siblings' sorting factors* |
| Genotypic correlation between full siblings | $f_{FS}=$ | $\frac{1+\mu(\tilde{a}_{1}+\tilde{c}_{1}\omega)^{2}}{2}$ | $f_{FS}=f_{DZ}$ |
| Focal Phenotype - Offspring Covariance   w/o assortative mating | $\lambda_{PO}=$ | $\frac{(a_{1}+c_{1}\omega)a'_{1}}{2}+(c_{1}+a_{1}\omega)c'_{1}+\sigma_{p}^{2}p$ | *Correlation between parental (focal) phenotype  and offspring phenotype* |
| Sorting Factor - Offspring Covariance   w/o assortative mating | $\lambda_{SO}=$ | $\frac{(\tilde{a}_{1}+\tilde{c}_{1}\omega)a'_{1}}{2}+(\tilde{c}_{1}+\tilde{a}_{1}\omega)c'_{1}+\sigma_{PS}p$ | *Correlation between parental sorting factor  and offspring phenotype* |
| **Cells in Covariance Matrix** | | | |
| ***Variances*** | | | |
| Observed Parental Phenotype | $\sigma_{p}^{2}=$ | $V_{A1}+V_{C1}+V_{T1}+V_{rGE1}+V_{E1}$ |  |
| Offspring Phenotype | $\sigma_{o}^{2}=$ | $V_{A1P}+V_{F}+V_{A2}+V_{C2}+V_{rGE2}+V_{E2}$ |  |
| ***Covariances in Parent Generation*** | | | |
| Siblings/Twins | $\delta_{PP}=$ | $fV_{A1}+r_{t}V_{T1}+V_{rGE1}+V_{C1}$ |  |
| Partners |  | $\sigma_{PS}^{2}\mu$ |  |
| Siblings-in-law |  | $\sigma_{PS}\mu\delta_{PS}$ |  |
| Co-siblings-in-law |  | $\sigma_{PS}^{2}\mu^{2}\delta_{SS}$ |  |
| ***Intergenerational Covariances*** | | | |
| Parent-Offspring |  | $\lambda_{PO}+\sigma_{PS}\mu\lambda_{SO}$ |  |
| Avuncular | $\tau=$ | $\frac{(fa_{1}+c_{1}\omega)a'_{1}}{2}+(c_{1}+a_{1}\omega)c'_{1}+\delta_{PP}p+\delta_{PS}\mu\lambda_{SO}$ | *The genetically related uncle/aunt* |
| Avuncular-in-law |  | $\sigma_{PS}\mu((\frac{(f\tilde{a}_{1}+\tilde{c}_{1}\omega)a'_{1}}{2}+(\tilde{c}_{1}+\tilde{a}_{1}\omega)c'_{1}+\delta_{PS}p)+\delta_{SS}\mu\lambda_{SO})$ | *The non-genetically related uncle/aunt* |
| ***Covariances in Offspring Generation*** | | | |
| Siblings |  | $f_{FS}a'_{1}^{2}+\frac{V_{A2}}{2}+V_{F}+V_{C2}+V_{rGE2}$ |  |
| Cousins |  | $\frac{fa'_{1}^{2}}{4}+qV_{A2}+\delta_{PP}p^{2}+c'_{1}^{2}+2p(c_{1}+a_{1}\omega)c'_{1}+p(fa_{1}+c_{1}\omega)a'_{1}+a'_{1}\omega c'_{1}+2\lambda_{SO}\mu(\frac{(f\tilde{a}_{1}+\tilde{c}_{1}\omega)a'_{1}}{2}+(\tilde{c}_{1}+\tilde{a}_{1}\omega)c'_{1}+\delta_{PS}p)+\lambda_{SO}^{2}\mu^{2}\delta_{SS}$ |  |
