# Supplementary material for: Understanding indirect assortative mating and its intergenerational consequences for educational attainment
Source: Nat Commun. 2025 Jun 6;16:5264. doi: 10.1038/s41467-025-60483-0 (PMC12144155; doi:10.1038/s41467-025-60483-0)
Supplement: Supplementary file 4 — Source Data [file 41467_2025_60483_MOESM4_ESM.zip › Source Data/supfig11.pdf]

a

Monozygotic Twin Families

	Twin 1	Twin 2	Partner 1	Partner 2	Child 1(1)	Child 2(1)	Child 1(2)	Child 2(2)
Twin 1		n = 2,447	n = 1,823	n = 1,862	n = 1,457	n = 1,444	n = 1,459	n = 1,449
Twin 2	.71		n = 1,823	n = 1,862	n = 1,457	n = 1,444	n = 1,459	n = 1,449
Partner 1	.48	.46		n = 1,238	n = 1,457	n = 1,444	n = 1,026	n = 1,017
Partner 2	.46	.52	.37		n = 1,038	n = 1,042	n = 1,459	n = 1,449
Child 1(1)	.39	.36	.36	.28		n = 1,078	n = 889	n = 868
Child 2(1)	.34	.33	.33	.24	.39		n = 877	n = 874
Child 1(2)	.36	.34	.26	.33	.24	.22		n = 1,046
Child 2(2)	.32	.30	.20	.34	.21	.17	.36	

b

Dizygotic Twin Families

	Twin 1	Twin 2	Partner 1	Partner 2	Child 1(1)	Child 2(1)	Child 1(2)	Child 2(2)
Twin 1		n = 3,360	n = 2,495	n = 2,488	n = 2,009	n = 1,976	n = 2,002	n = 1,991
Twin 2	.45		n = 2,495	n = 2,488	n = 2,009	n = 1,976	n = 2,002	n = 1,991
Partner 1	.45	.32		n = 1,623	n = 2,009	n = 1,976	n = 1,330	n = 1,347
Partner 2	.30	.47	.28		n = 1,349	n = 1,337	n = 2,002	n = 1,991
Child 1(1)	.34	.22	.33	.19		n = 1,490	n = 1,133	n = 1,135
Child 2(1)	.33	.24	.34	.18	.36		n = 1,103	n = 1,138
Child 1(2)	.22	.32	.17	.36	.18	.15		n = 1,505
Child 2(2)	.21	.33	.22	.33	.16	.17	.35	

c

Full Sibling Families

	Sibling 1	Sibling 2	Partner 1	Partner 2	Child 1(1)	Child 2(1)	Child 1(2)	Child 2(2)
Sibling 1		n = 206,263	n = 206,263	n = 206,263	n = 171,558	n = 171,664	n = 171,545	n = 171,556
Sibling 2	.41		n = 206,263	n = 206,263	n = 171,558	n = 171,664	n = 171,545	n = 171,556
Partner 1	.47	.29		n = 206,263	n = 171,558	n = 171,664	n = 171,545	n = 171,556
Partner 2	.29	.47	.25		n = 171,558	n = 171,664	n = 171,545	n = 171,556
Child 1(1)	.35	.23	.34	.18		n = 136,959	n = 143,529	n = 143,516
Child 2(1)	.34	.23	.34	.18	.36		n = 143,546	n = 143,674
Child 1(2)	.23	.35	.18	.34	.17	.17		n = 136,838
Child 2(2)	.23	.34	.18	.34	.17	.17	.36	
