# Supplementary material for: Understanding indirect assortative mating and its intergenerational consequences for educational attainment
Source: Nat Commun. 2025 Jun 6;16:5264. doi: 10.1038/s41467-025-60483-0 (PMC12144155; doi:10.1038/s41467-025-60483-0)
Supplement: Supplementary file 4 — Source Data [file 41467_2025_60483_MOESM4_ESM.zip › Source Data/Table_ACEfit.docx]

| **Supplementary Table: Testing parameters in the iAM-ACE model** | | | | | | | |
| --- | --- | --- | --- | --- | --- | --- | --- |
| **Base** | **Comparison** | **Parameters** | **-2LL** | **df** | ***Δ*LL** | ***Δ*df** | ***p*** |
| + Stratification |  | 10 | 2,247,740 | 845,324 |  |  |  |
| **+ Stratification** | **Full iAM-ACE model^a^** | **9** | **2,247,740** | **845,325** | **0.0** | **1** | **9.997 × 10^-1^** |
| Full iAM-ACE model | Direct Assortment + Measurement Error | 7 | 2,248,277 | 845,327 | 536.9 | 2 | 2.611 × 10^-117^ |
| Full iAM-ACE model | Direct Assortment | 6 | 2,256,338 | 845,328 | 8,598.2 | 3 | 0 |
| Full iAM-ACE model | No Assortment (*μ* = 0) | 5 | 2,359,308 | 845,329 | 111,567.6 | 4 | 0 |
| ^a^Reported parameters are from this model | | | | | | | |
