# Supplementary material for: Understanding indirect assortative mating and its intergenerational consequences for educational attainment
Source: Nat Commun. 2025 Jun 6;16:5264. doi: 10.1038/s41467-025-60483-0 (PMC12144155; doi:10.1038/s41467-025-60483-0)
Supplement: Supplementary file 4 — Source Data [file 41467_2025_60483_MOESM4_ESM.zip › Source Data/Table_Correlations.docx]

| **Supplementary Table: Correlations (95% Confidence Intervals)** | | | |
| --- | --- | --- | --- |
| **Relation** | **Monozygotic** | **Dizygotic** | **Full Sibling** |
| Siblings/Twins | .706   *(.696, .715)* | .450   *(.428, .470)* | .407   *(.404, .410)* |
| Partners | .504   *(.485, .523)* | .460   *(.442, .477)* | .465   *(.463, .467)* |
| Siblings-in-law | .462   *(.441, .483)* | .310   *(.286, .334)* | .292   *(.289, .295)* |
| Co-Siblings-in-law | .371   *(.332, .410)* | .274   *(.234, .312)* | .249   *(.245, .253)* |
| Parent-Offspring | .341   *(.321, .361)* | .333   *(.317, .350)* | .341   *(.339, .343)* |
| Avuncular | .340   *(.318, .362)* | .223   *(.200, .245)* | .228   *(.226, .231)* |
| Avuncular-in-law | .247   *(.216, .277)* | .183   *(.156, .211)* | .178   *(.176, .181)* |
| Siblings (Offspring) | .370   *(.339, .400)* | .355   *(.328, .381)* | .356   *(.353, .359)* |
| Cousins (Offspring) | .216   *(.179, .252)* | .161   *(.128, .194)* | .168   *(.165, .171)* |
