# Supplementary material for: Understanding indirect assortative mating and its intergenerational consequences for educational attainment
Source: Nat Commun. 2025 Jun 6;16:5264. doi: 10.1038/s41467-025-60483-0 (PMC12144155; doi:10.1038/s41467-025-60483-0)
Supplement: Supplementary file 4 — Source Data [file 41467_2025_60483_MOESM4_ESM.zip › Source Data/Table_COTSfit.docx]

| **Supplementary Table: Testing parameters in the iAM-COTS model** | | | | | | | |
| --- | --- | --- | --- | --- | --- | --- | --- |
| **Base** | **Comparison** | **Parameters** | **-2LL** | **df** | ***Δ*LL** | ***Δ*df** | ***p*** |
| **Full iAM-COTS model^a^** |  | **17** | **4,090,739** | **1,545,428** |  |  |  |
| Full iAM-COTS model | *a*_1_′ = 0 | 16 | 4,090,936 | 1,545,429 | 196.8 | 1 | 1.052 × 10^-44^ |
| Full iAM-COTS model | *c*_1_′ = 0 | 16 | 4,090,752 | 1,545,429 | 12.8 | 1 | 3.492 × 10^-4^ |
| Full iAM-COTS model | *p* = 0 | 16 | 4,090,786 | 1,545,429 | 46.4 | 1 | 9.651 × 10^-12^ |
| Full iAM-COTS model | Direct Assortment + Measurement Error | 15 | 4,091,254 | 1,545,430 | 514.3 | 2 | 2.068 × 10^-112^ |
| Full iAM-COTS model | Direct Assortment | 14 | 4,100,038 | 1,545,431 | 9,299.0 | 3 | 0 |
| ^a^Reported parameters are from this model | | | | | | | |
