# Supplementary figures and images for: Understanding indirect assortative mating and its intergenerational consequences for educational attainment
Source: Nat Commun. 2025 Jun 6;16:5264. doi: 10.1038/s41467-025-60483-0 (PMC12144155; doi:10.1038/s41467-025-60483-0)

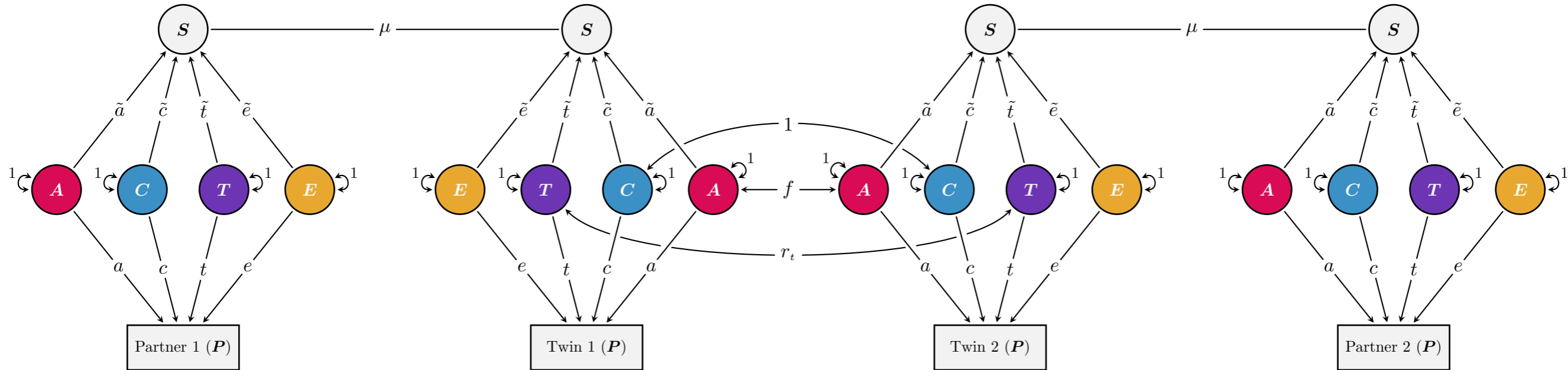

Supplement: Supplementary file 4 — Source Data [file 41467_2025_60483_MOESM4_ESM.zip › Source Data/fig3.pdf]

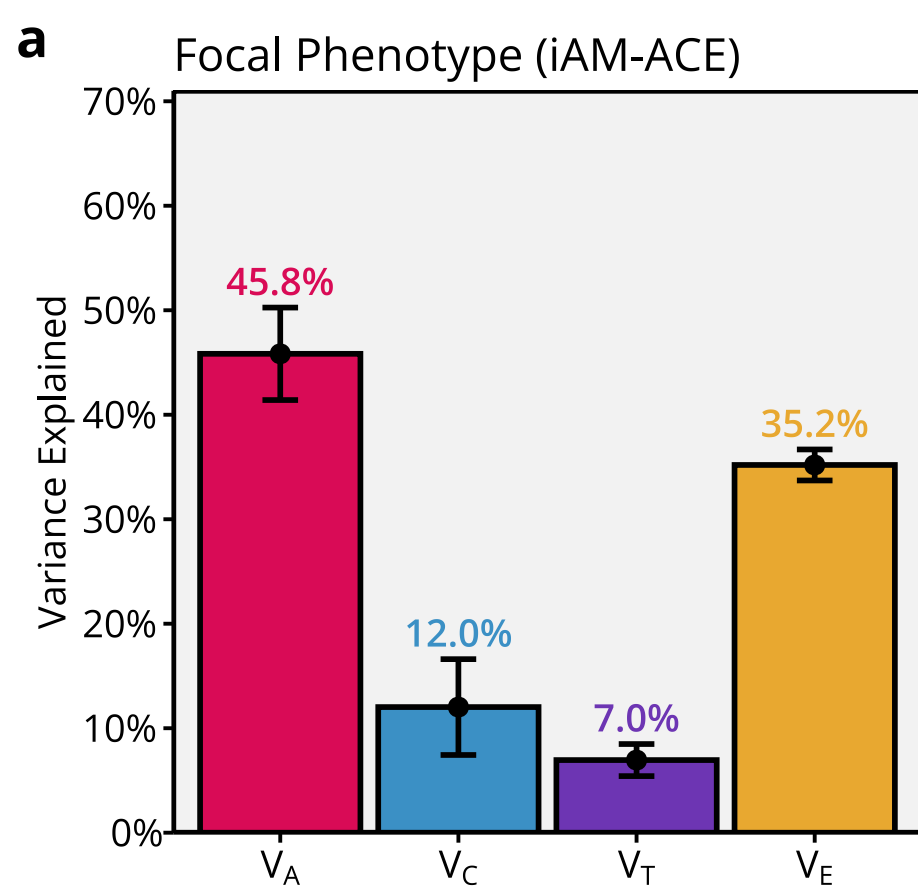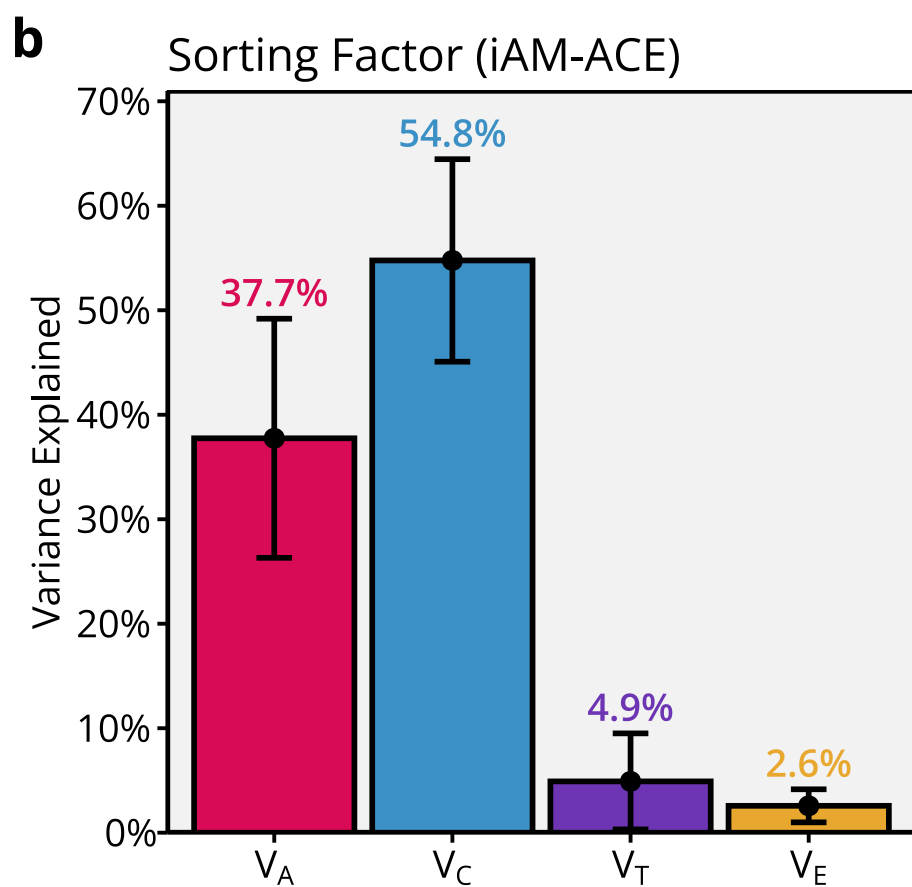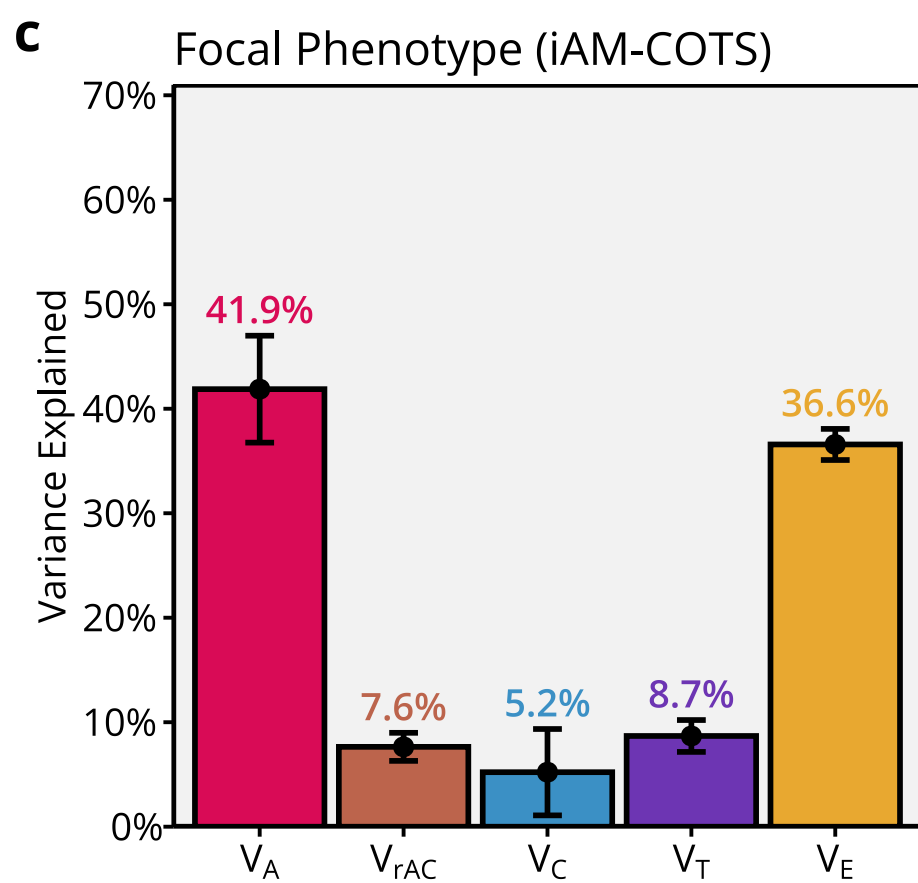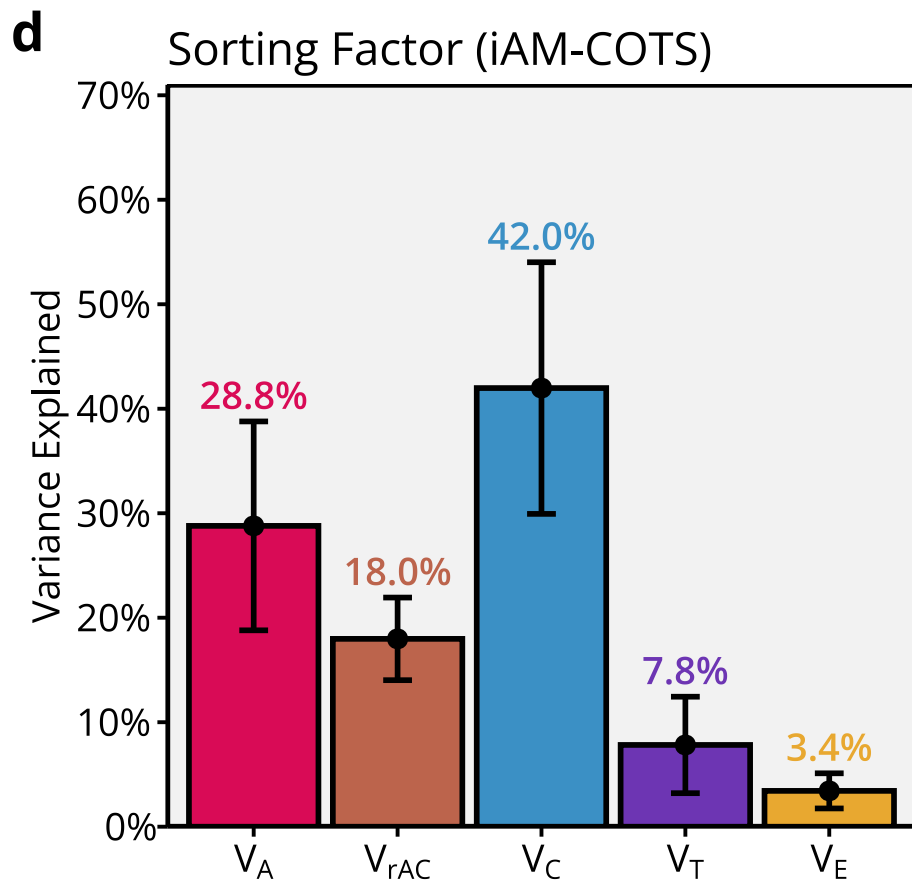

Supplement: Supplementary file 4 — Source Data [file 41467_2025_60483_MOESM4_ESM.zip › Source Data/fig4.pdf]

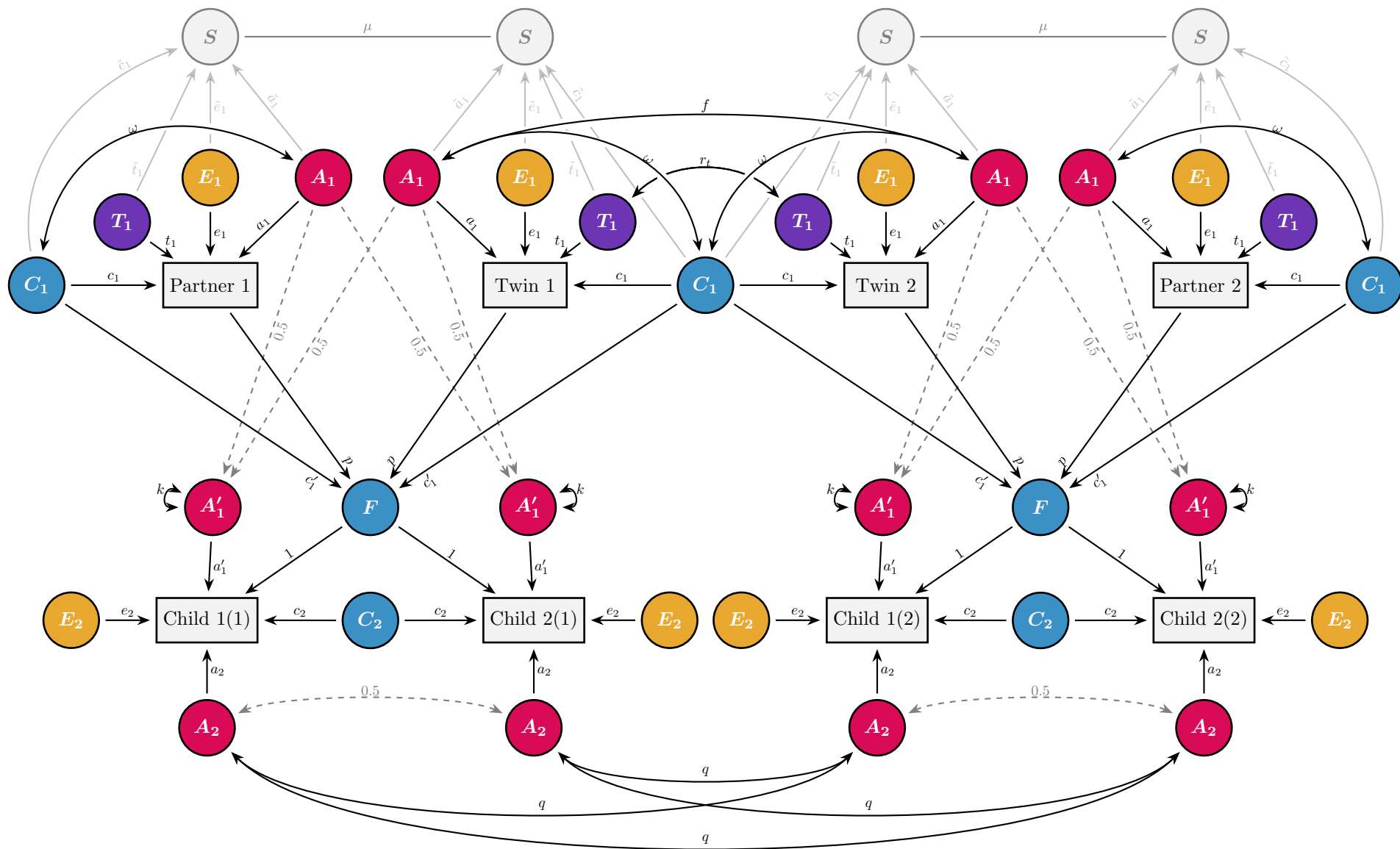

Supplement: Supplementary file 4 — Source Data [file 41467_2025_60483_MOESM4_ESM.zip › Source Data/fig5.pdf]

**a**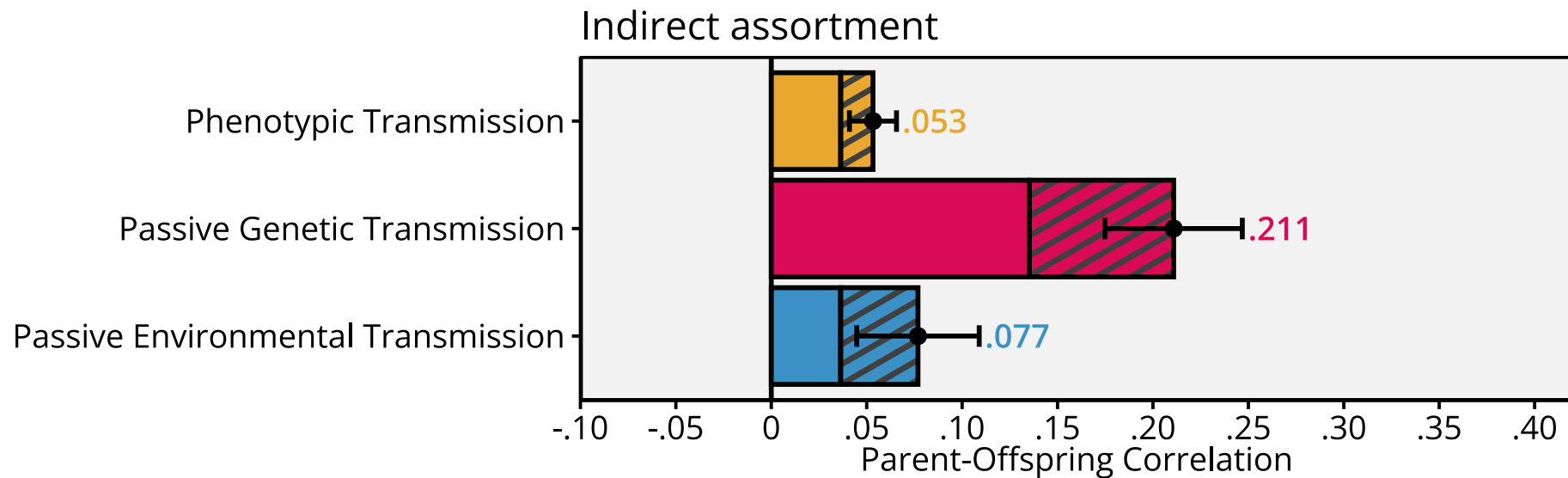**b**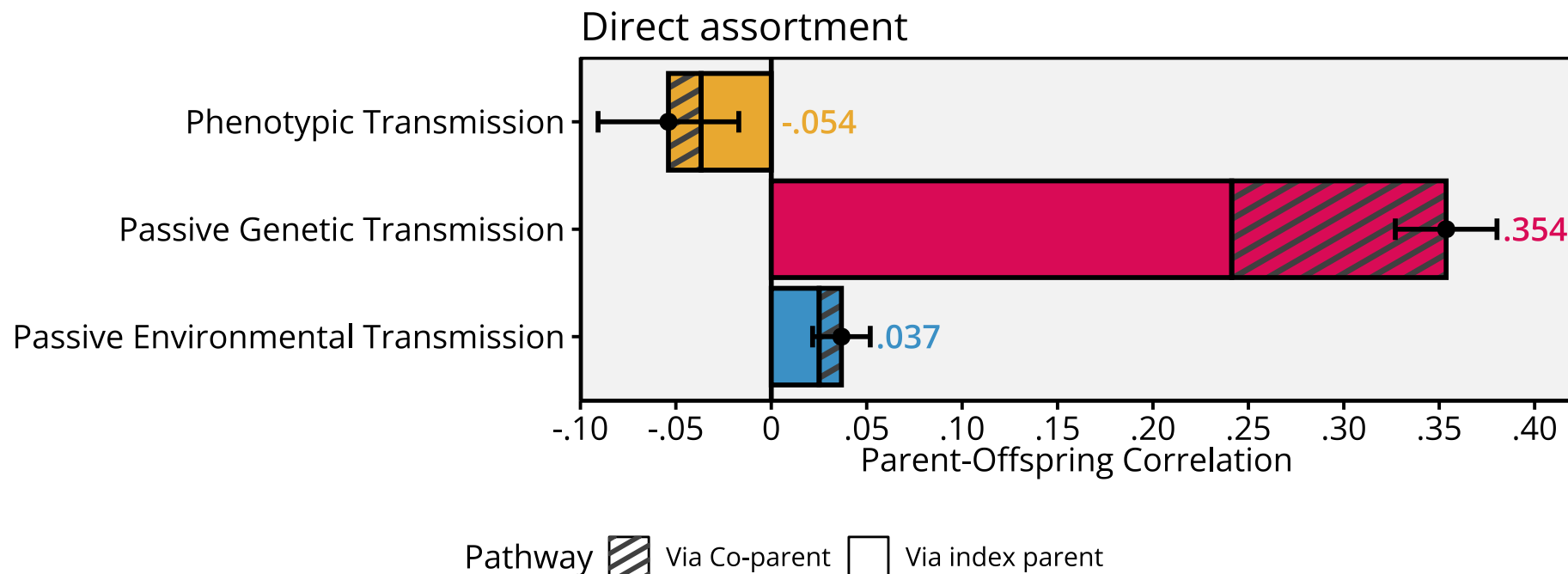

Supplement: Supplementary file 4 — Source Data [file 41467_2025_60483_MOESM4_ESM.zip › Source Data/fig6.pdf]

# Offspring Educational Attainment

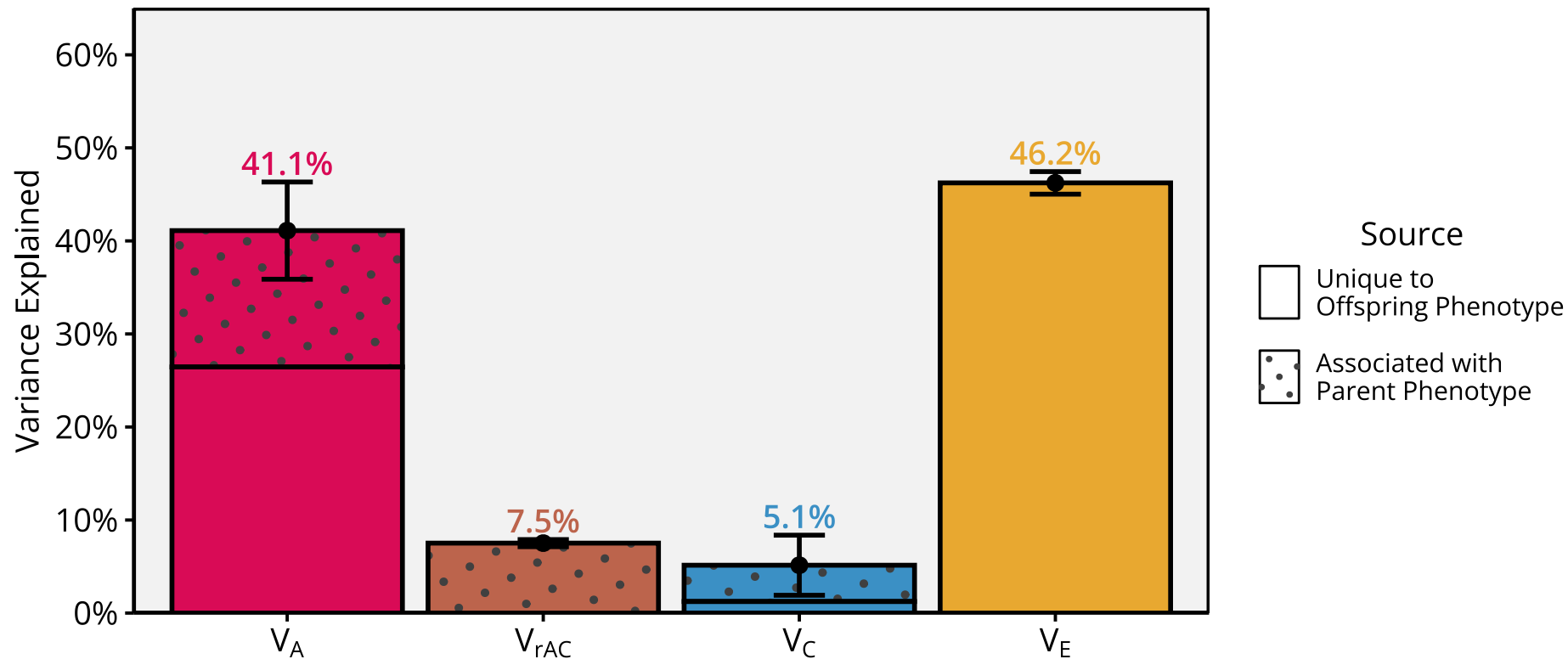

Supplement: Supplementary file 4 — Source Data [file 41467_2025_60483_MOESM4_ESM.zip › Source Data/fig7.pdf]

**a** Focal Phenotype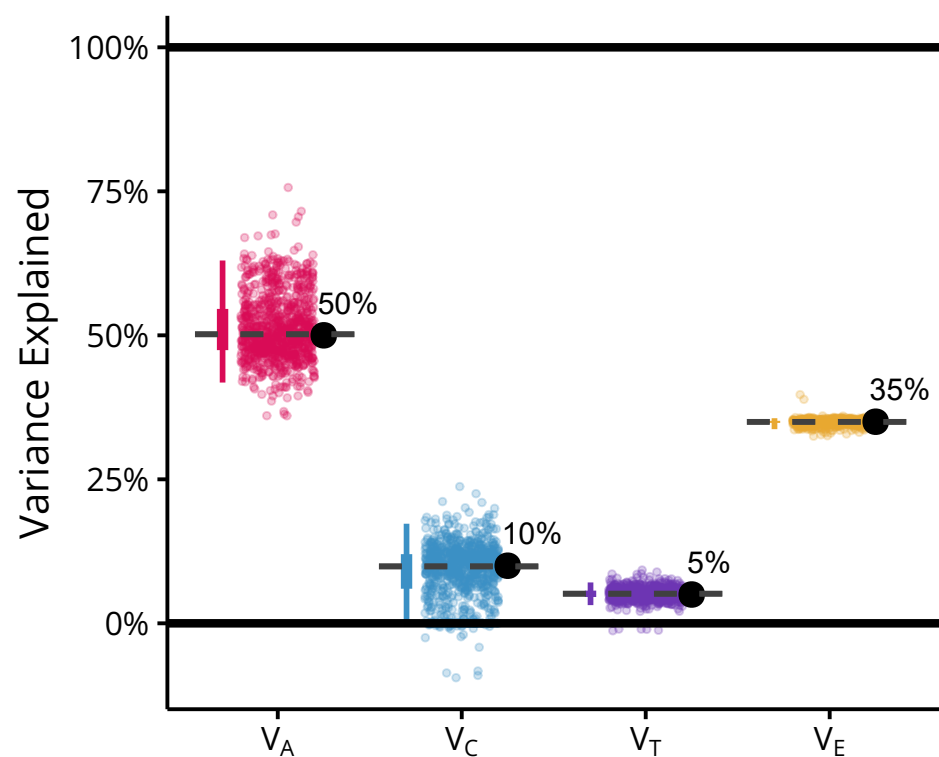**b** Sorting Factor Partner Correlation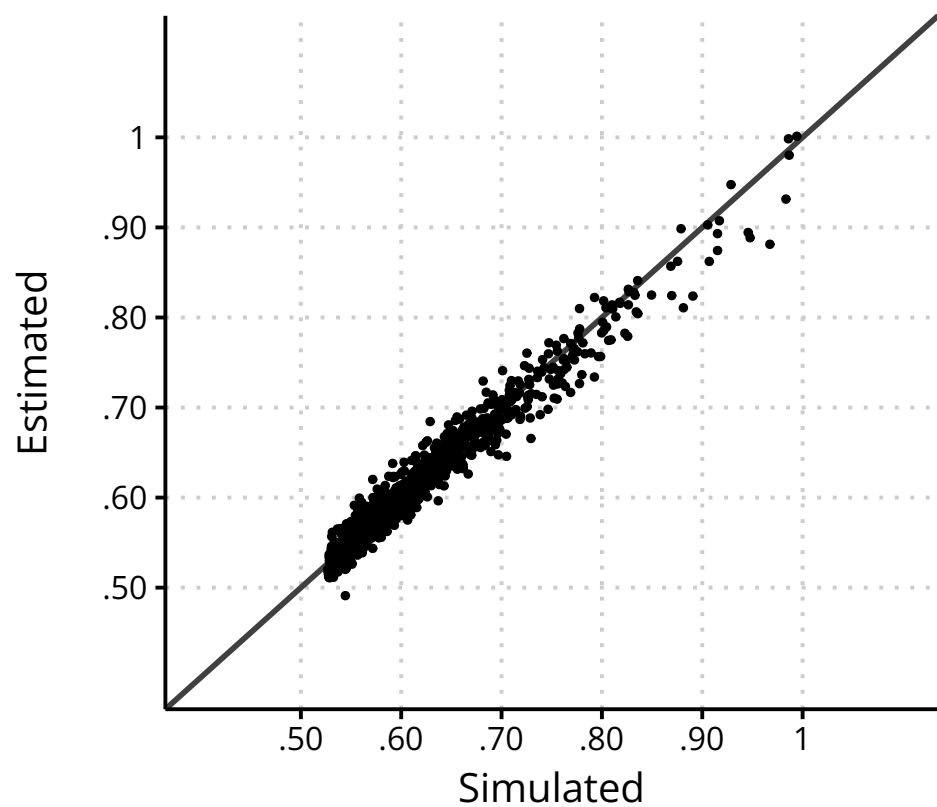**c** Genetic Homogamy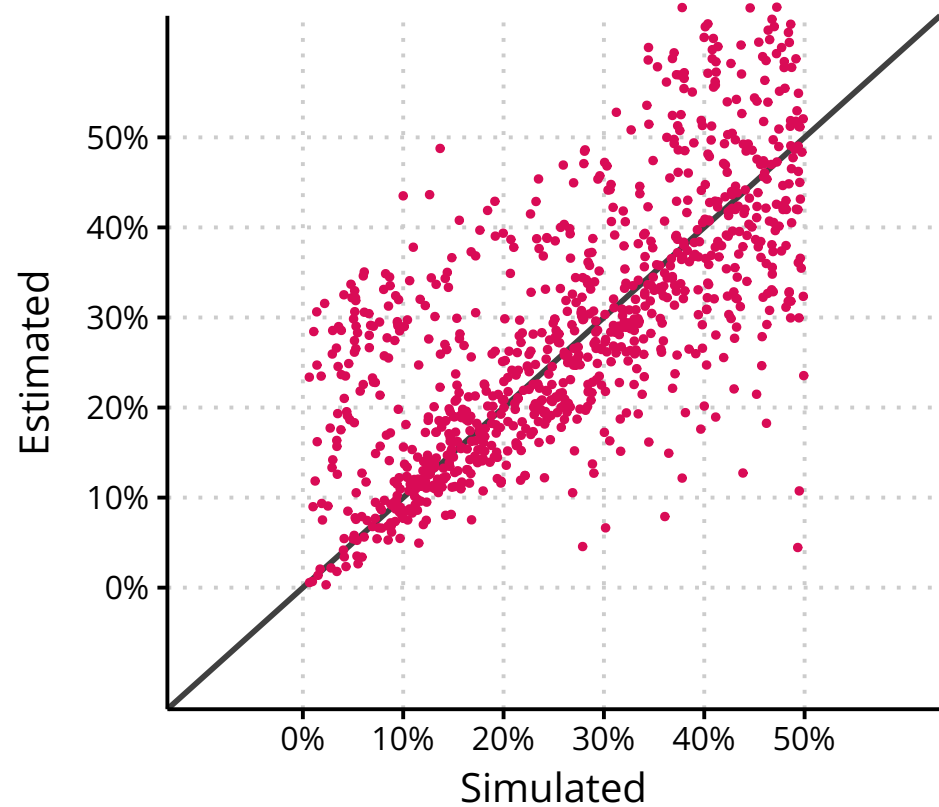**d** Social Homogamy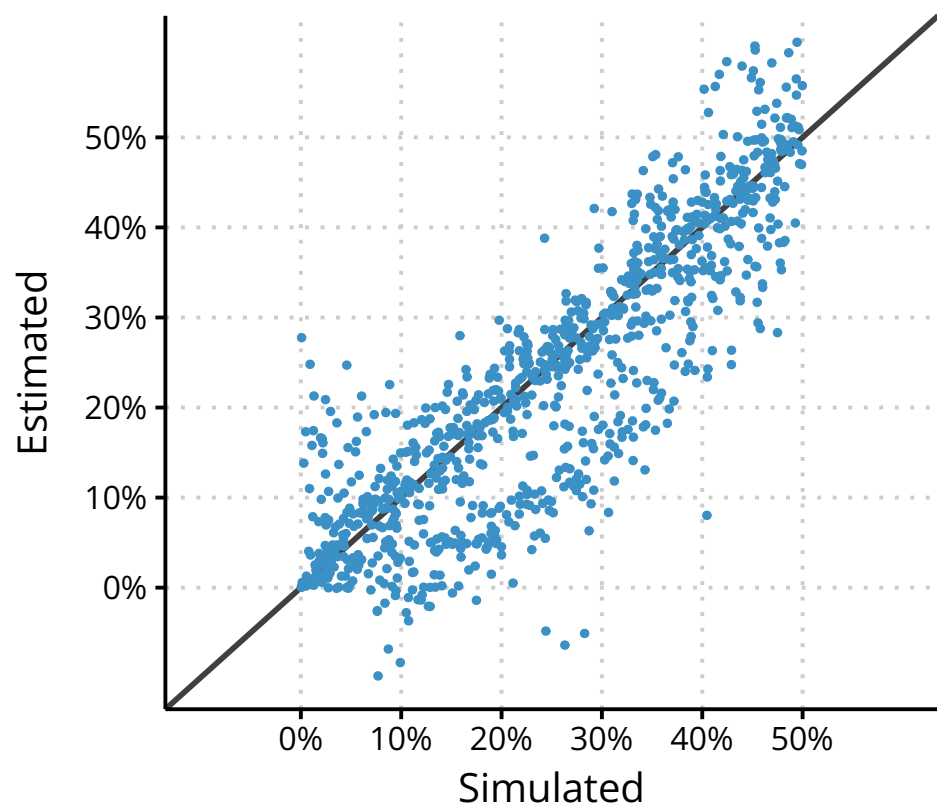

Supplement: Supplementary file 4 — Source Data [file 41467_2025_60483_MOESM4_ESM.zip › Source Data/Simulations/acesim_fig.pdf]

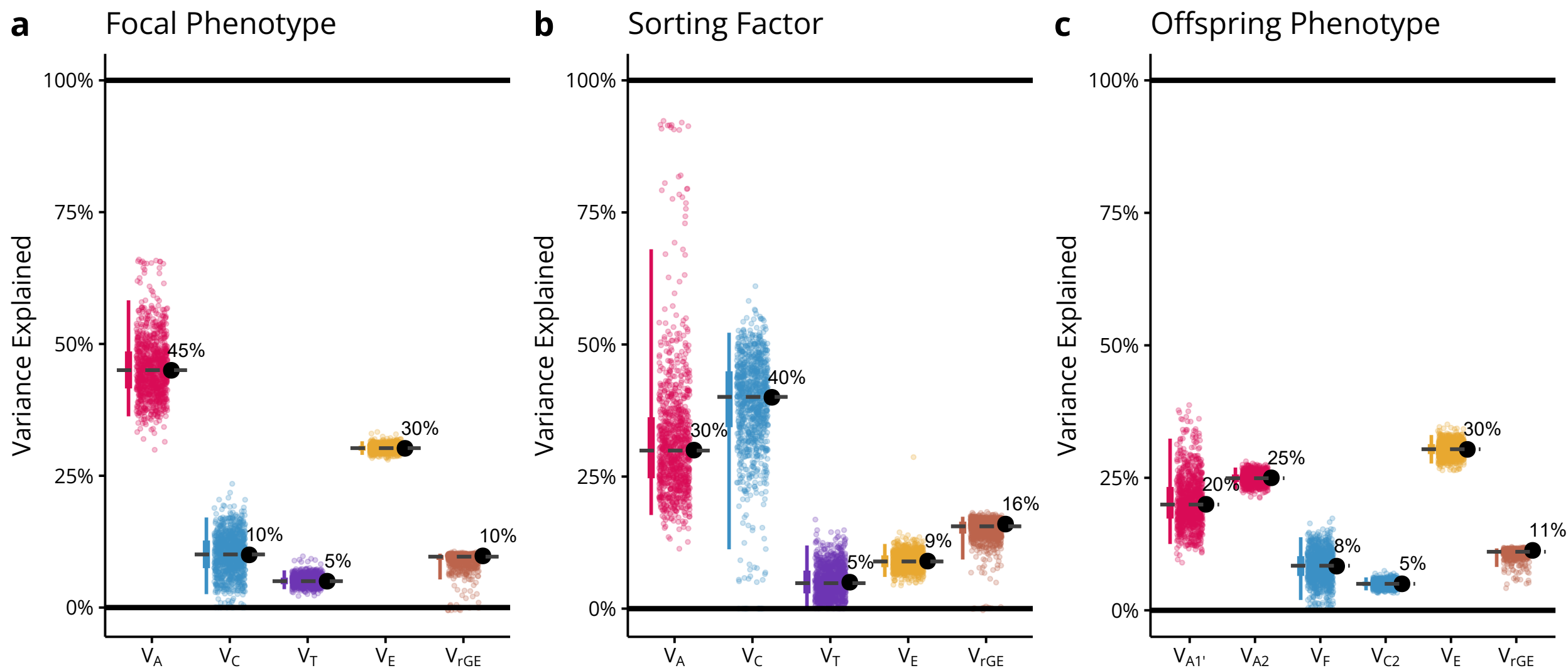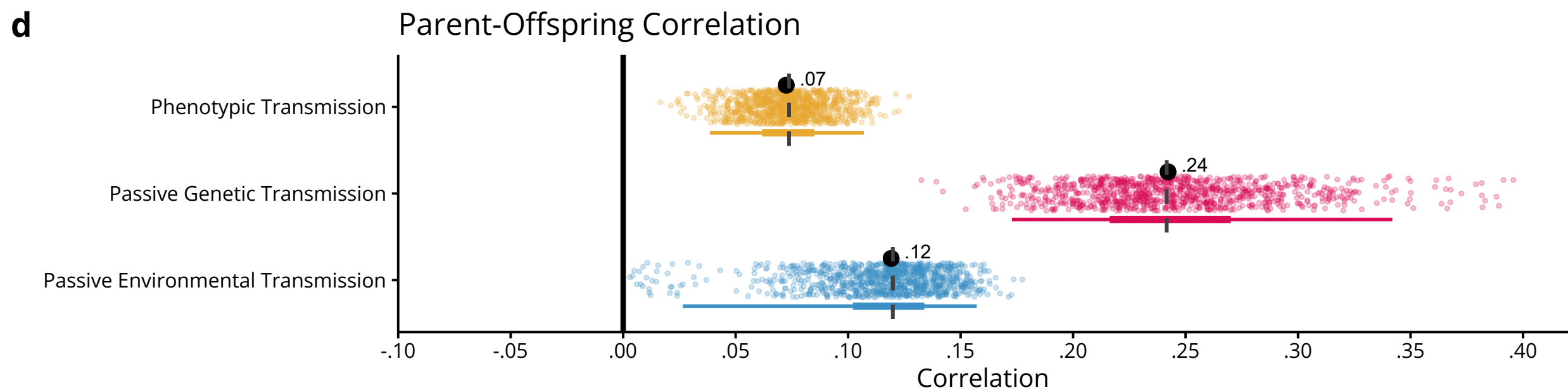

Supplement: Supplementary file 4 — Source Data [file 41467_2025_60483_MOESM4_ESM.zip › Source Data/Simulations/cotssim_fig.pdf]

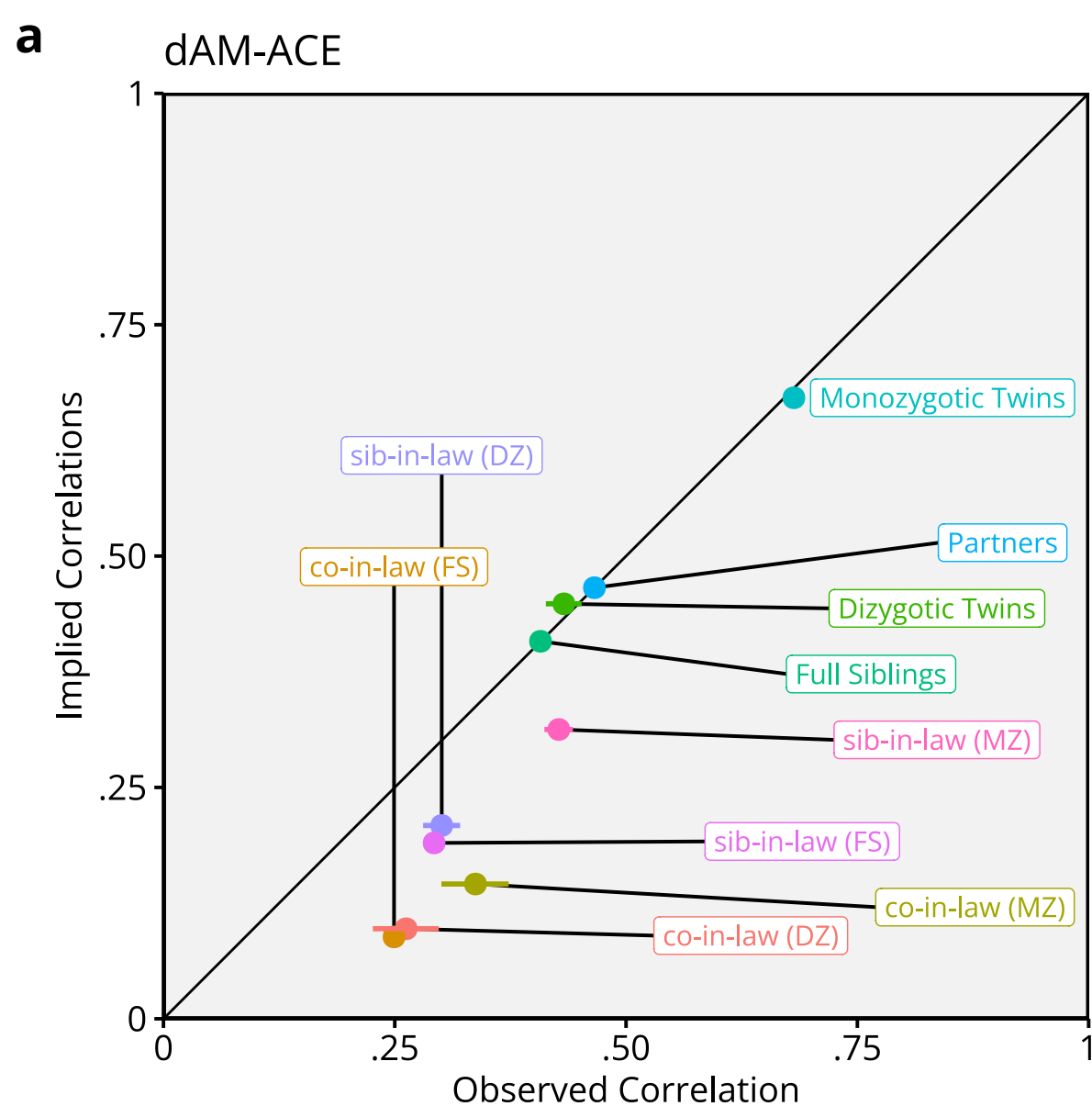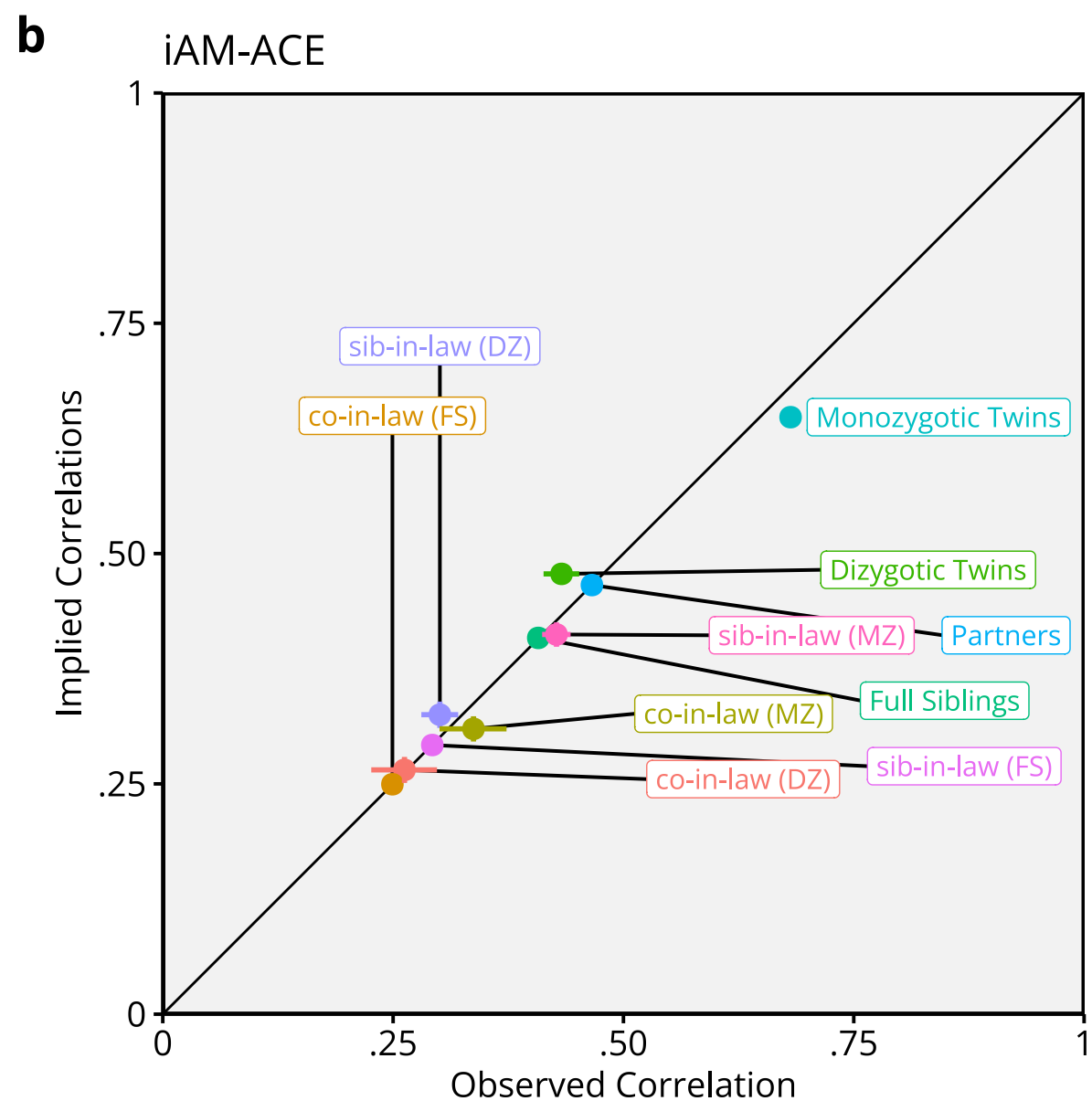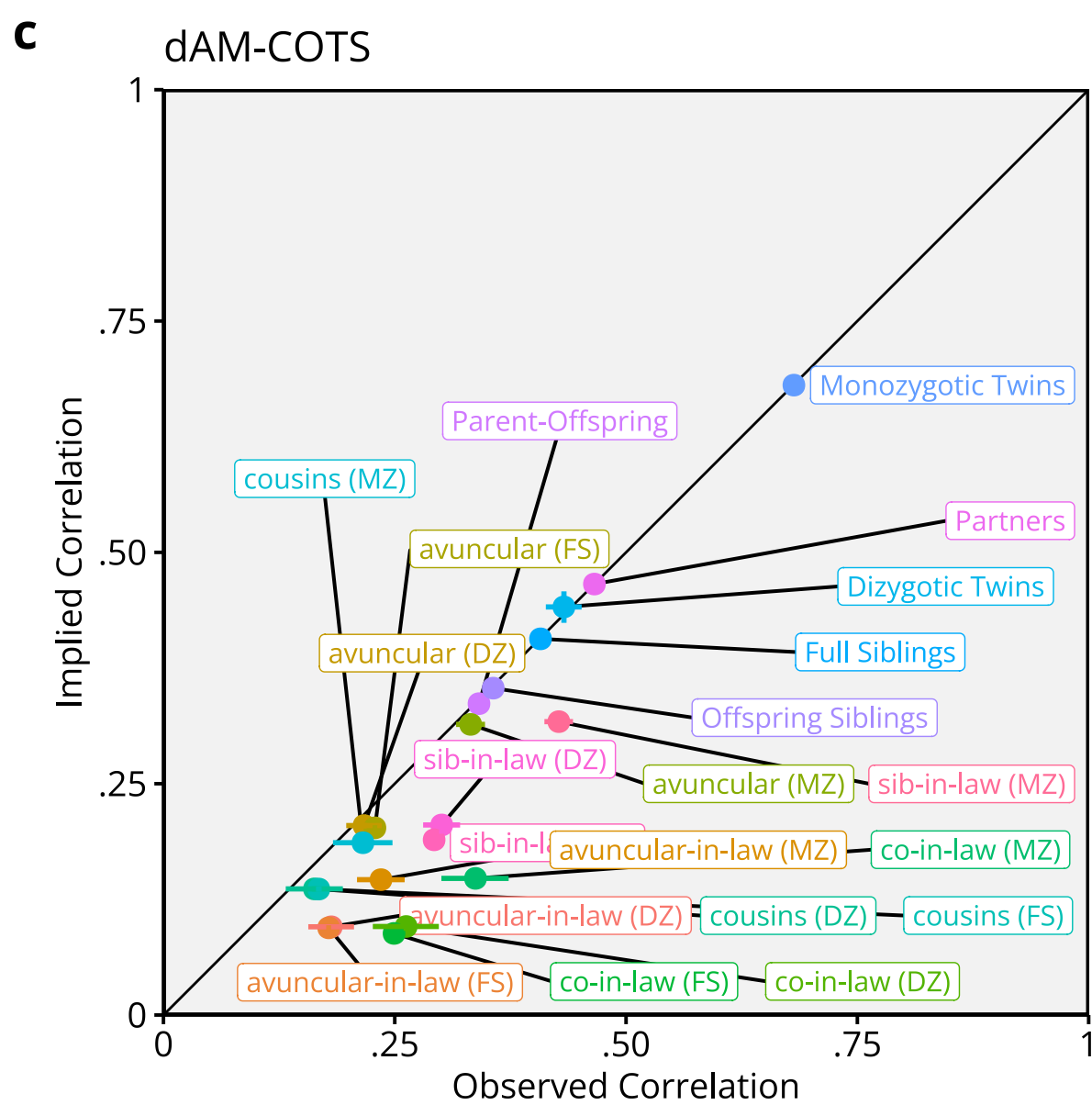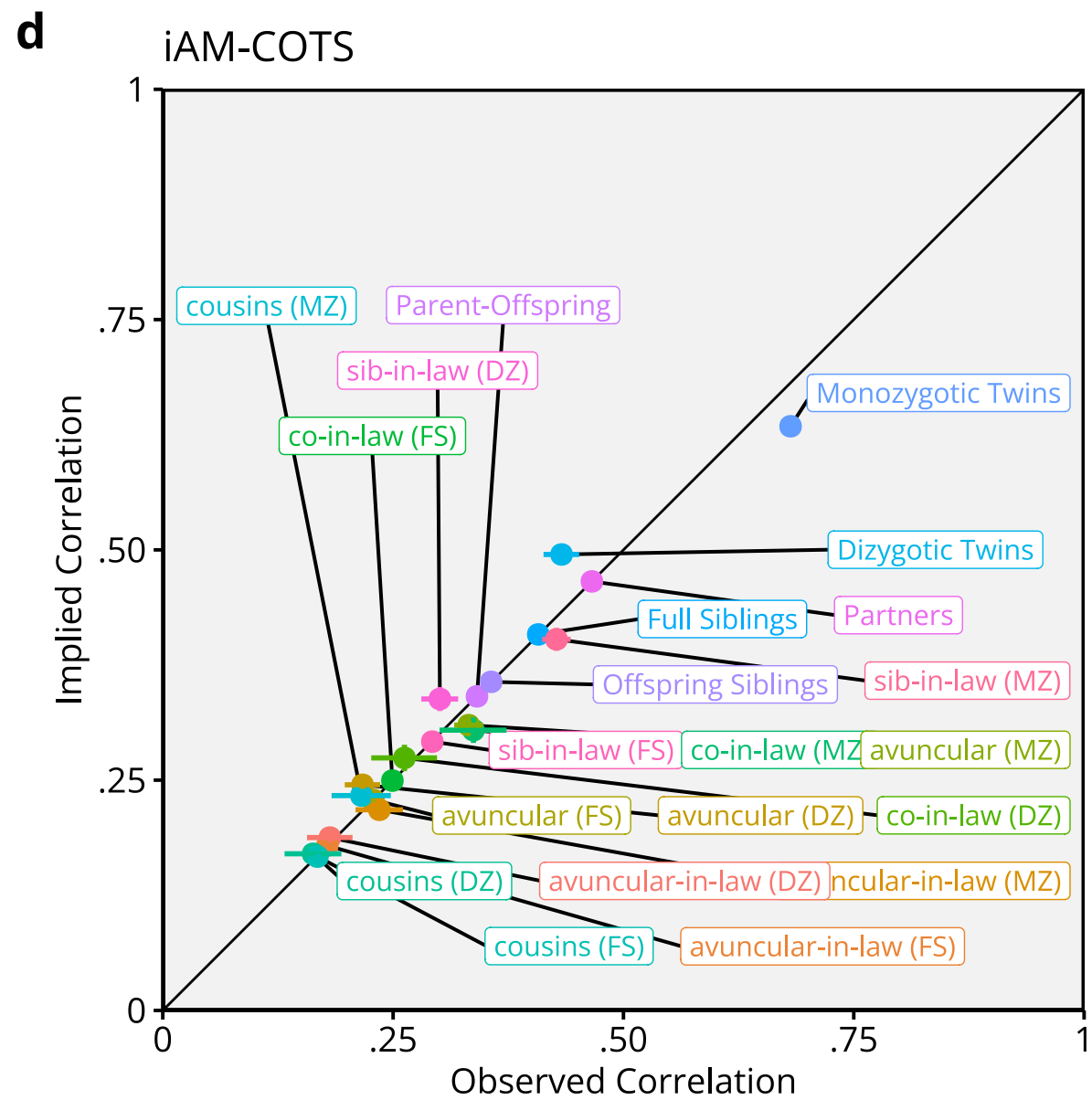

Supplement: Supplementary file 4 — Source Data [file 41467_2025_60483_MOESM4_ESM.zip › Source Data/supfig12.pdf]

**a**

iAM-ACE

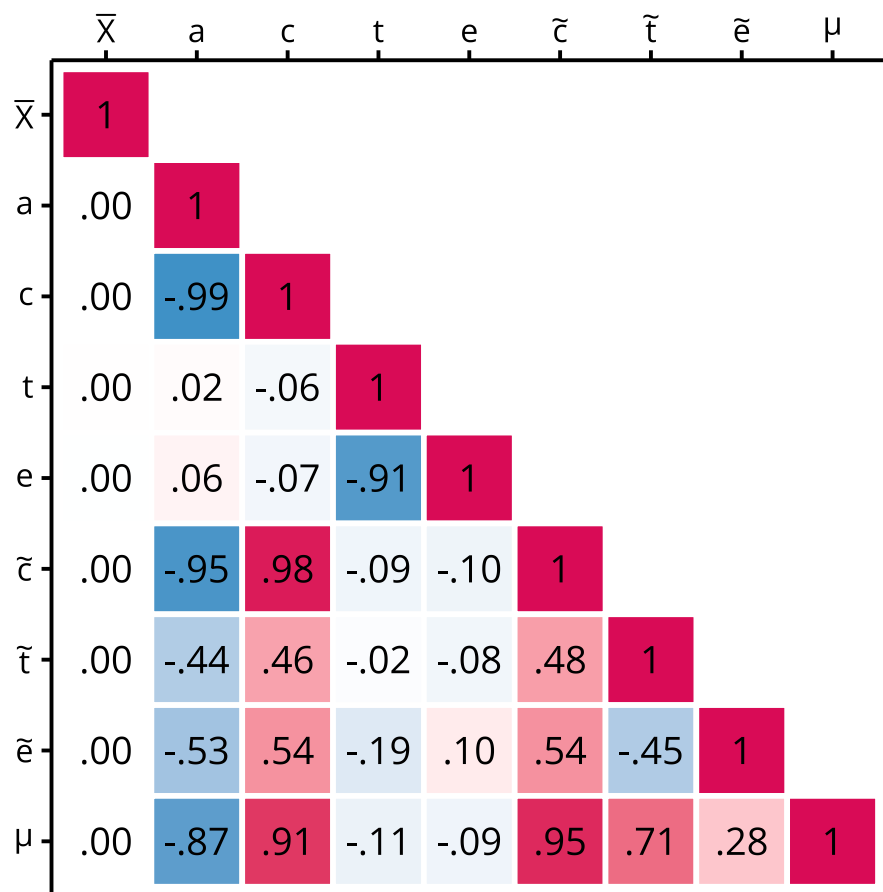**b**

iAM-COTS

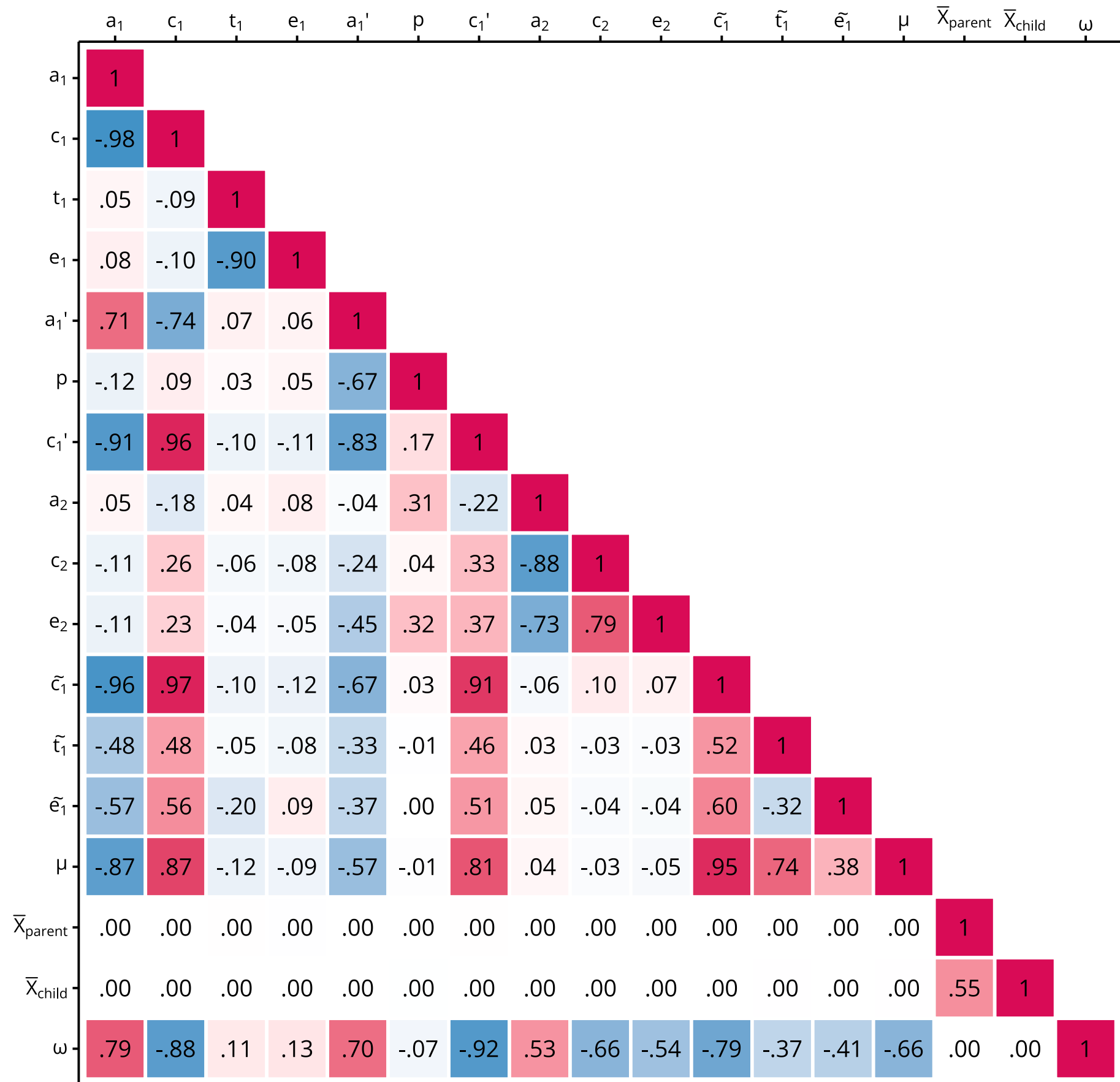

Supplement: Supplementary file 4 — Source Data [file 41467_2025_60483_MOESM4_ESM.zip › Source Data/supfig13.pdf]
