# Supplementary material for: Understanding indirect assortative mating and its intergenerational consequences for educational attainment
Source: Nat Commun. 2025 Jun 6;16:5264. doi: 10.1038/s41467-025-60483-0 (PMC12144155; doi:10.1038/s41467-025-60483-0)
Supplement: Supplementary file 3 — Transparent Peer Review file [file 41467_2025_60483_MOESM3_ESM.pdf]

nature portfolio

Peer Review File

Understanding indirect assortative mating and its intergenerational consequences for educational attainment

Corresponding Author: Dr Hans Fredrik Sunde

This file contains all reviewer reports in order by version, followed by all author rebuttals in order by version.

Version 0:

Reviewer comments:

Reviewer #1

(Remarks to the Author)
Thanks for the opportunity to read the manuscript: “Understanding indirect assortative mating and its intergenerational consequences”. The paper aims – and succeeds - to bring more clarity to cloudy ideas surrounding genetic and social (in)direct Assortative Mating (AM). I find this an excellent study that I believe the literature would benefit greatly from. I have only one major point to discuss and a couple of minor mentions and suggestions.
Major:
In line 61, the authors state that: “For example, assortment on heritable traits leads to genetic similarity between partners.” I have two comments regarding this statement. First, in particular after reading this study – but also before – I believe this is not true. There are two scenarios I would imagine given my current understanding in which assortment on a heritable trait might happen but there is no genetic correlation. The first scenario would be depicted in Fig 1 e, social stratification. If there is no mating preferences, but only environmental factors are responsible for trait similarity between mates – would there be increased genetic similarity? In the second scenario – and this is related – there is only a non-genetic correlation between the sorting trait and the assorted, heritable phenotype. This is not very realistic but possible. I would challenge the authors to consider these scenarios with potentially zero inflated genetic similarity between partners.

My understanding would be that the authors even hint on this in their model of added complexity on AM in line 150f: “For example, the genetic consequences only depend on how much the genetic influences on the focal phenotype are assorted upon, regardless of whether assortment is direct or not.:

And l 155f: “Note that the sorting factor only includes the components of the associated traits that are associated with the focal phenotype.”

Minor
- L 27: “Understanding indirect assortment is crucial for resolving the role of nature and nurture in education.”
This sounds a bit vague for the general motivation of this research.

- L 51 – typo: "progress have been hampered"
Should be “has”?

- L 163f: “… Idiosyncratic homogamy for the degree to which non-shared environmental influences on the focal phenotype are associated with the sorting factor ($\tilde{e}$in Figure 1b).”
Could the authors provide a reasoning behind coining the non-shared environmental model “idiosyncratic”?

- I’ve had been puzzle where the authors subsume structural/selection effects such as higher interaction density amongst highly educated due to University attendance which results in AM independent of preferences. This is discussed in detail but it seems subsumed under the social stratification model. The social stratification model, however, only focusses on C but such an effect can also emerge in E. I would prefer a general discussion of structural – also geographic effects – in a separate selection model. Selection versus preferences are discussed in respect to AM in detail in l 208f but I would prefer a separate model regarding this and the authors should note that disentangling structure/selection effects from preferences/homogamy is a larger discussion in the sociological literature for sure, see e. g.:
Skopek, J., Schulz, F., & Blossfeld, H. P. (2011). Who contacts whom? Educational homophily in online mate selection.

European Sociological Review, 27(2), 180-195.

- I think it would be great if “indirect assortative mating” is defined in Table 1.

- When the authors discuss the results in e. g. l 246f: “When accounting for this in intergenerational models, we found that 62% of the parent-offspring correlation could be explained by passive genetic transmission, whereas the remaining was explained by direct (15%) and passive (23%) environmental transmission.”, it might help the reader to mention the bias in the models not considering indirect AM explicitly since these models are the innovation.

- I got confused with the numbers of samples. The authors state there are: “1,529,144 individuals in 209,792 extended families”, but then mention “2,407 244 monozygotic twins, 3,330 dizygotic twins, and 204,055 full siblings” as in 209,792 individuals. Which number is correct for individuals and families?

- The authors state in l 254 that participants are born “…1975 and 1995 (and thereby old enough to have obtained education themselves).” It is fine that they are old enough that they had the opportunity to obtained education but to avoid right censoring they all should be beyond the observation window that allows still be in education - if that exists. However, it is realistic to still be in education when 29 years old nowadays. How did the authors deal with individuals still in education – e. g. a PhD.

- In l 329 testing for measurement error, it says: “This resulted in significantly worse 330 fit than the full model” I was wondering why the authors term the model without measurement error “full model”.

- The authors nicely demonstrate the utility of their extended framework in twin studies. I would really like to see a more extensive conceptual discussion how this impacts molecular genetically informed research and how PGSs can be used more felixible in this context etc.

- Overall, I really like this study and would wish these authors would write a similar paper on pleiotropy.

Reviewer #2

(Remarks to the Author)
review by CVDolan of
Understanding indirect assortative mating and its intergenerational consequences by Hans Fredrik Sunde, Espen Moen Eilertsen, Fartein Ask Torvik.
Addresses the process underlying assortatitve mating (a.m.). This is an important subject generally, and a highly relevant subject given the present strong interest in genetic influences on socially relevant phenotypes, notable educational attainment (i.e., the rise of sociogenomics), and the possibly implications for social inequality, which characterizes Western societies. Includes a useful discussion of the often muddy terminology, and a conceptually explicit outline of possible processes, with explicit definitions. That is very useful. Includes an excellent application to educational attainment age age 30 in an impressive Norwegian data set (about 210.000 families, about 1.530.000 individuals). The application involves two designs: the iAM-ACE and iam-COTS, where the latter is better, but comes greater data requirements. I have comments, given below. Textual (minor) comments are given below (separate header).
My general impression is that this is well-executed important work, and that this paper will become highly influential, given its comprehensive approach to a.m.
Comments
My general comment is that a lot of information is presented in the suppl. But I think some details could be included in the main text (if feasible, given word number limitations of the journal). See below.
This paper focuses largely on EA. Should this be reflected in the title? The process giving rise to assortative mating, presumably, depends on the phenotype under consideration. Would it be useful to be explicit in this regard?
The paper considers EA, and positive assortment. That could be stated, so that other statements do not raise the issue of the sign of the spousal correlation (e.g.,217 “Under assortative mating, the correlation between a parent and their offspring will in most cases be inflated,” i.e., positive assortment?)
138 Figure 1 and 278. The term “copath” features twice in the text. I think that it would be useful to present this (if not 138, then 278) with a little more explanation, as it is a technical terms. E.g., identify it explicitly as the correlation that arises from the assortment process (matching on the sorting variable) in so far as it is manifest in the phenotype of interest.
“Finally, by defining homogamy with respect to the177 focal phenotype, consequences of assortative mating can be derived without involving global statements178 about partner formation”.
Does this mean: without having to identify the process of partner formation, beyond the role of the sorting factor and its relationship with the causes of the phenotype (i.e., sources of phenotypic variance)?
“This can come about from some form of190 assortative mating as described above, where covariance is induced without affecting the variance” This is an important point which could be presented more forcefully in the preceding text (& Fig 1).
174 more generally, where the degree of genetic, social, and idiosyncratic homogamy is equal to the relative173 importance of genetic, social, and idiosyncratic factors on the focal phenotype ($a = \tilde{a}$, $c = \tilde{c}$, and $e = \tilde{e}$).

Here we see equalities. But later on the term proportionality is used (see line 230). Proportionality is more general than equality. So should a proportionality model be used here, rather than equalites? I think I that the phenotypic variance is not constrained, but the factor S is standardized, implying a~^2 + c~^2 + e~^2 = 1 (or later in iAM-ACE; a~^2 + c~^2 + e~^2 + t~^2 = 1). But if this is so, the $a = \tilde{a}$, is not likely ever to hold.
198 different mechanism compared to assortment on social characteristics31
See also https://link.springer.com/article/10.1007/BF01065887
For example,202if potential partners cared about the socioeconomic position of their would-be parents-in-law, then this would203 be assortative mating.
should be: "…this would be social homogamy"?
222 "Likewise, if a parent directly influences their children's222 traits, the other parent is likely to exert a similar influence, thus inflating the parent-offspring correlation."
I do not understand the stipulation / specification here that the other parent exert a similar influence.
Cohorts are from 1975 to 1995. The correlations (fig 2) express the straightforward phenotypic relations, not fixed covariates, I assume?
The presentation of the models in the results section is as per journal requirement? The models are not really results.
Figure 2. Possible to include the 95CI numerically next to the correlation (fig 2)? E.g., (say)
Siblings/Twins … n=3300 | r=.44 (.41 - .47).
Figure 2. It would be helpful perhaps to add to the figure caption examples of the relationships, so that it clear e.g., Avuncular-inlaw.
292 "This would imply a genotypic correlation between partners of .26 (.22, .29)." It would be instructive to add the calculation leading to .26. I assume that that can be done in a single sentence. Also, it would be useful to report how the phenotypic spousal correlation is decomposed into the latent component (A + the others).
319 The test of phenotypic (direct) assortment fails, as it to be explected given the previous results. The test is one of " equality" constraints. As mentioned, I assume a~^2 + c~^2 + e~^2 + t~^2 = 1 so 3 df is understandable. The exact way the equality (or proportionality) in implemented could be mentioned in the main text, with ref to the relevant suppl. Later on the model is relaxed to estimate e~^2 freely. Again how this is done could be mentioned in the main text, again one or two sentences.
338. The term "gene-environment correlation" is general. This could be expressed in terms of the actaul latent variables in the model, which would be more precise: I assume it is A – C covariance.
344 "Because children of monozygotic twins will be as related to their parent as their parent's twin, children of 344 twins can be used to decompose the parent-offspring correlation into parts attributable to genetic 345 transmission and various forms of environmental transmission". Please state the "forms of environmental transmission" with meaning in natural language.
Consider: 489 etc. " Our results 489 suggest that genetic similarity can account for much of this correlation (62%), but that a substantial part must 490 be ascribed to various forms of environmental transmission." Important results, do statement of what the various forms are seem important to explicate.

Minor textual. Not necessarily important: FYI.
59 "Assortative mating occurs when individuals with similar traits mate more often than expected by chance." "similar traits". Ambiguous? I suppose that the phenotypic manifestation of assortative mating is the phenotypic correlation among partners. So assortative mating implies a non-zero phenotypic correlation among partners. "Similar traits" is strange, as everyone has these traits. Assortment is the similarity, spousal / partner correlation traits of interest.
63 "Assortative mating has important consequences for intergenerational transmission and will often bias63 estimates unless accounted for properly11,13,19." At this point is it not clear: estimates of what? Perhaps add…. often bias estimates of, for instance X, Y, Z, unless accounted …
75 "Numerous lines of evidence suggest that direct assortment is an inadequate explanation for partner similarity in educational attainment, and that partners must instead be more highly correlated on an associated variable."
The comparative: more highly, more highly than what? "… and that the phenotypic correlation among partners must be based on one or more associated variables." It seems too restrictive to assume "an associated variable", as the assortment may be based on two or more variables.
78 "Robinson, et al. 6 found that education-associated genetic similarity between partners in the UK Biobank78 implied a correlation between partners in the assorted variable of .65, much higher than observed the79 phenotypic correlation for educational attainment at .41."
I assume: … implied an EA attainment correlation between the partners of .65". The variation is in terms: "education-associated" vs "eductional attainment". So the education-associated variable is EA?
89 "For example, Kemper, et al. 23 attempted to infer the89 phenotypic correlation between spouses' educational attainment by investigating phenotypic correlations90 between distant relatives in the UK Biobank."
"attempted" is ambiguous. "… Kemper, et al. inferred the ….?
93 "Similarly, Clark investigated correlations in social class among distant relatives in another large, English data set and found that genetic similarity alone could account for most of the familial resemblance if it were the case that partners correlated .79 on a highly heritable unobserved factor.
I lost the connect here with direct phenotypic assortment. I understand that the genetic similiarity is inconsistent with direct phenotypic assortment. But here a "highly heritable unobserved factor" is introduced. This factor is the direct assortment variable? But here the phenotype is social class. See also 95 ff: "The unobservable trait associated with EA".
220 "For example, if partners are genetically similar, then offspring will tend to inherit genetic220 variants with similar effects from both parents, thus inflating the genetic similarity between a parent and their221 offspring (and hence also phenotypic similarity)12.
I find the additional "with similar effects" hard to follow. The effect of the genetic variant is given (ruling out age x gene interaction).

226 Models assuming direct assortment, such as most extended twin models with partners, implicitly226 assume that all pathways are inflated to the same degree, proportional to the phenotypic correlation between227 partners. Perhap mention what pathways you are referring to. I assume that this related to line 174.
233 There may be numerous ways to estimate the induced correlations in causes without assuming direct234 assortative mating.
“may be” is vague: There are numerous ways…

Reviewer #3

(Remarks to the Author)
The paper by Sunde et al. “Understanding indirect assortative mating and its 7 intergenerational consequences” has many important contributions and insights into the phenomenon of assortative mating (in human) populations. The ambition of the paper is to develop a new framework, define key terms and apply the a new model to data on educational attainment from Norway.
As indicated, the paper has valuable elements, but I could not help that the claims might be too broad, that the treatment is not as fully comprehensive as suggested and that too much ignored from earlier work by others.

For example: line 43/44: “Mating partners tend to have similar educational attainment”: this statement is based on a single population, i.e. UKB which not only has an overrepresentation of more highly educated (female) participants but also comes from a country with a class system that is more culturally ingrained than in many other countries. The difference between the UK and Sweden for example (lines 88-102) could also explain the differences in the results as noted? The authors acknowledge that cultural differences may exist (between UK and Norway -see discussion- but deem these unlikely as an explanation for differences in results?).

The sentence “Researchers broadly agree..”: I am not sure that this is backed up by references 2-8.
Maybe similar, the references 9-14 include books that are so broad (e.g. an intro textbook of behavior genetics) that is hard to see how a reader can understand why it is important correctly model assortment in order to understand social inequality.

Line 59: assortative mating (AM) occurs ...: maybe ‘describes would be better?
There are multiple consequences of assortative mating for traits influenced by genetics, including greater homozygosity for loci related to these traits. Over generations, this can amplify genetic similarities within subgroups of a population, even leading to reduced genetic diversity within subgroups, but in the population as a whole to an increase in genetic variation (see e.g. Falconer: Introduction to Quantitative Genetics). A focus on bias in parameter estimates (line 64) may be important, but does not seem a ‘consequence of AM’? (see also the paragraph beginning at line 216).

Line 64/65: Most prior .... I am not sure this is true and would have expected a reference to e.g. Phillips et al. (DOI: 10.1007/BF01260935) who proposed to model multivariate assortment in order to test for the presence of an indirect marital correlation (in their case between education in one spouse and intelligence in the other), testing if the correlation is due only to the direct pairing on education and on intelligence and to the within-person correlation between the two traits.

Introduction of models: why is genetics / genotype reduced to additive genetic influences? In a plea for rigorous terms, I would hope to see a treatment of the full genotype (A+D). Also, I struggle with the definition of family-shared environment: suppose partner 1 has a sib-environment with multiple brothers and sisters whereas their partner is from a single offspring family. They cannot assort on the shared environment? What happens to the variance induced by the shared environment?

Line 477 and further: the summary of the results of other studies does not seem entirely adequate (e.g. Gonggrijp et al. conclude “both social homogamy and phenotypic assortment are important processes in spouse selection in Finland and the Netherlands. In both countries, phenotypic assortment contributes to a greater degree to the similarity of spouses than social homogamy”).

Please let my observations not distract from the fact that there are many valuable points and results in this paper. I would ask the authors to adjust their phrasing.

Version 1:

Reviewer comments:

Reviewer #1

(Remarks to the Author)
Thank you for the opportunity to read the revised version of the manuscript 'Understanding indirect assortative mating and its intergenerational consequences'. The reviewers have thoroughly addressed all my comments and I would like to see this paper as part of the literature. I have only one outstanding comment: We engaged in a discussion on whether the are at 30 is the end of the observation window for educational attainment, and it turned out that some of the folks are even only observed until they are 27 years old. I would like to see this as part of the discussion section as a limitation of the empirical study.

Other than that, I would like to congratulate the authors for their great work.

Reviewer #2

(Remarks to the Author)
Review by CVDolan of the revision of
"Understanding indirect assortative mating and its intergenerational consequences"
bby Hans Fredrik Sunde1*, Espen Moen Eilertsen2, Fartein Ask Torvik1,2

The authors have adequately addressed my previous points. My subsequent comments concerns mainly textual edits. I am happy to leave it to the authors whether to act on these or not. Line numbers are including in the quoted text (identifying the location in the ms). I have a few substantive comments (indicated with the ***). I imagine that these can be addressed easily. I consider this an excellent paper, truly "a steppingstone in …the right direction". The fact that these models require huge sample sizes does not detract from the conceptual and theoretical analysis of the issue of assortment.

"Mating partners tend to have similar educational attainment1. Researchers broadly agree that this results from38 assortative mating on associated traits rather than, or in addition to, education itself, as … "

Interpunction? Suggestion:

Mating partners tend to have similar educational attainment1. Researchers broadly agree that this results from assortative mating on associated traits, in addition to possible assortment on education attainment itself, as …

"More specifically, it is when partner formation is partly conditional on individuals' having similar trait56 values, resulting in covariance between partners' traits."

More specifically, it arises when …

"on the questionable assumption that assortment takes place73 directly on the observed phenotype"

Arguably questionable in a study of educational attainment. But not generally questionable. Therefore: "on the strong assumption that …. "

"Under direct assortment, the expected genotypic correlation76 Understanding indirect assortative mating […] 2 between partners is known, and"

"is known"? Does this not require additional information? Can be estimated? If I say the mating is due to direct assortment and the spousal phenotypic correlation is .3, you do not know the genotypic correlation (as it depends on the other parameters in the model, plus all the assumptions about "interplay").

"However,78 matching on a phenotype will also lead to partner correlations on associated phenotypes28."

Here you are still referring to direct assortment. If so please state. "However, direct assortmnet on a phenotype…"

"that partners must instead be more highly correlated similar on an associated87
variables."

ambiguous comparative: more similar than what? The message is that the assortment is on traits related to EA, I think.

106 "Hindrances for understanding of indirect assortative mating"
Hindrances to ….

"For example, social homogamy is often implied to mean122 assortment on cultural factors only27,32."

It would perhaps be useful to explicate "cultural factors". Ambiguous term ? What are these? I assume "environmental" (given the text later on: "model environmental similarity between family members, and thereby precluded a cultural explanation"). Anyway: "cultural factors" is an ambiguous term. This is dealt with later on. It might help the reader to state that fact.

*** Fig 1: I like the clear representation of competing models. We see that A and C are uncorrelated in model 1b (the model of interest here) and we see that A and C are uncorrelated in 1e. I wonder whether it is desirable at this point to state explicitly this aspect of these model, and whether the presence of AC covariance has any bearing. This is specifically of interest in the case of AE, which presumably is subject to AC cov. Intergenerational equilibrium may hold with respect to the genetic nature process. I suppose that this is dealt with in the second model (see line 391). Maybe useful to alert the reader to this here?

"Only the relative importance of $\tilde{a}$, $\tilde{c}$,$\tilde{t}$, and $\tilde{e}$ can be estimated, meaning the variance of the sorting factor must be348 constrained." Indeed this was mentioned earlier "To make the model identified, we constrained the variance of the sorting factor313 to equal the phenotypic variance."

So perhaps this could be mentioned explicitly in figure caption (248).

*** “To assume direct assortment, the degree of genetic,362 social, and individual assortment idiosyncratic homogamy must be constrained to equal their importance for363 the focal phenotype ($a = \tilde{a}$, $c = \tilde{c}$, $t = \tilde{t}$, and $e = \tilde{e}$), which makes the focal phenotype and the sorting364 factor identical. This removes four parameters and one constraint, giving three fewer degrees of freedom. We365
find that constraining the model to direct assortment results in substantially worse fit (Δ -2LL = 8590.0, Δdf366 = 3, p < .001, Supplementary Figure 12)”.
We read that that var(S) = var(focalPh), i.e., an identification constraint. The parameters $\tilde{a}$, $\tilde{c}$, $\tilde{t}$, and $\tilde{e}$, are free parameters initially. The equality constraints ($a = \tilde{a}$, $c = \tilde{c}$, $t = \tilde{t}$, and $e = \tilde{e}$) implies + 4 df. So I assume that once these constraints are in place, the constraint var(S) = var(focalPh) is no longer necessary, so that implies -1 df resulting in +3 df? It would be useful to state explicitly “This removes four parameters and allows us to relax the constraint that the variance of S equals the variance of the focal phenotype”. It this is correct, are the models actually nested (justifying the use of the LRT)? I understand that
model A: ($a = \tilde{a}$, $c = \tilde{c}$, $t = \tilde{t}$, and $e = \tilde{e}$) and var(Ph) = var(S)
is nested under
model B: (a != $\tilde{a}$, c != $\tilde{c}$, t != $\tilde{t}$, and e != $\tilde{e}$) and var(Ph) = var(S)
But is
model A: ($a = \tilde{a}$, $c = \tilde{c}$, $t = \tilde{t}$, and $e = \tilde{e}$) and var(Ph) = var(S)
nested under
model C: ($a = \tilde{a}$, $c = \tilde{c}$, $t = \tilde{t}$, and $e = \tilde{e}$) and var(Ph) != var(S)
?
If is presumably possible to step down from B to A (4 df) to C (1 df). Here the substantive meaning of the comparison A vs C requires explication. see also line 381 and lines 432-433.

“we found that 62% of the479 parent-offspring correlation could be explained by passive genetic transmission, whereas the remaining was480 explained by direct (15%) and passive (23%) environmental transmission”
“….of the phenotypic parent-offspring correlation…” be explicit?

*** I wonder how high the ML parameter estimates are actually correlated in these two models (I think this info can be extracted uing the vcov() function.

Reviewer #3

(Remarks to the Author)
The authors have done a responsible job in responding to reviewers. My main struggle with their work is that is ‘confined’ to their own framework (‘the framework we present’) which employs refined definitions of common terminology. For example, AM is not AM when it cause is social stratification. Many researchers will define any resemblance between spouses as AM. There are many more examples of ‘under definitions’. NB see response to R3 (line 59): this seems to imply that any spouse resemblance maybe called AM.

The motivation for the paper -modified in abstract- is to gain understaning on intergeneration transmission: could the authors add a conclusion after the analysis of the impressive dataset, about what we learned regarding this form of transmission? As the second aim of the paper (resposne to R2) is to study EA, this should be reflected in title.
EA is a trait with high spouse correlations, which as not seen for most other traits. How much would a reseracher gain by applying this new framework to other traits?

Version 2:

Reviewer comments:

Reviewer #2

(Remarks to the Author)
I thank the authors for their careful and detailed response to my earlier comments (second review).
I have no further comment on this version. Congrats to the authors on an excellent and important paper.

Open Access This Peer Review File is licensed under a Creative Commons Attribution 4.0 International License, which permits use, sharing, adaptation, distribution and reproduction in any medium or format, as long as you give appropriate credit to the original author(s) and the source, provide a link to the Creative Commons license, and indicate if changes were made.
In cases where reviewers are anonymous, credit should be given to 'Anonymous Referee' and the source.
The images or other third party material in this Peer Review File are included in the article's Creative Commons license, unless indicated otherwise in a credit line to the material. If material is not included in the article's Creative Commons license and your intended use is not permitted by statutory regulation or exceeds the permitted use, you will need to obtain permission directly from the copyright holder.
To view a copy of this license, visit https://creativecommons.org/licenses/by/4.0/

Response to reviewers

We have made revisions to our manuscript in response to the reviewers' comments. In addition to issues highlighted by the reviewers, we have made a few changes in response to comments received from others outside the review process. First, we have added Supplementary Figure 12, which compares observed and implied correlations under direct and indirect assortment in the two models (ACE and COTS). Second, we have changed confidence intervals to Wald type as we suspected the original bootstrapped confidence intervals were artificially narrow. The confidence intervals are therefore wider now. All other changes in the main manuscript are mentioned below next to the relevant comment (except typos).

**Reviewer #1's comments**	**Our response and relevant changes**
Thanks for the opportunity to read the manuscript: "Understanding indirect assortative mating and its intergenerational consequences". The paper aims – and succeeds - to bring more clarity to cloudy ideas surrounding genetic and social (in)direct Assortative Mating (AM). I find this an excellent study that I believe the literature would benefit greatly from. I have only one major point to discuss and a couple of minor mentions and suggestions.	We thank the reviewer for their overall positive appraisement and their thorough and helpful comments, which we address below.
**Major:**<br>In line 61, the authors state that: "For example, assortment on heritable traits leads to genetic similarity between partners."<br>I have two comments regarding this statement. First, in particular after reading this study – but also before – I believe this is not true. There are two scenarios I would imagine given my current understanding in which assortment on a heritable trait might happen but there is no genetic	We thank the reviewer for this interesting challenge. We believe the statement is true in the framework we present (we explain why below), but because the truth of this statement is not evident in the context in which it is written, and because it comes before we present the refined definitions, we have revised the phrasing:<br><br>*Line 62:*<br>When mating is conditional on specific traits, the causes of these traits and (other associated factors) will become correlated across partners. For example, if the traits are associated with genetic differences, partners will tend to be genetically similar.

correlation. The first scenario would be depicted in Fig 1 e, social stratification. If there is no mating preferences, but only environmental factors are responsible for trait similarity between mates – would there be increased genetic similarity? In the second scenario – and this is related – there is only a non-genetic correlation between the sorting trait and the assorted, heritable phenotype. This is not very realistic but possible. I would challenge the authors to consider these scenarios with potentially zero inflated genetic similarity between partners.<br><br>My understanding would be that the authors even hint on this in their model of added complexity on AM in line 150f: "For example, the genetic consequences only depend on how much the genetic influences on the focal phenotype are assorted upon, regardless of whether assortment is direct or not.:<br><br>And l 155f: "Note that the sorting factor only includes the components of the associated traits that are associated with the focal phenotype."	(Note that the preceding definition of assortative mating has also been revised in response to several comments made by Reviewer #2).<br><br>**As for whether the statement is true:**<br>The original statement "*assortment on heritable traits leads to genetic similarity between partners*" hinges on what we mean by "*assortment*" and what we mean by "*on heritable traits*". We would argue that the first scenario presented by the reviewer is not "assortment", and the second scenario does not involve assortment "on heritable traits". Let us explain in more detail:<br><br>*<u>Scenario 1 (Social Stratification):</u>*<br>As the reviewer notes, under social stratification, partner similarity is caused by shared prior causes (e.g., identical environments), rather than by matching on traits. This scenario does not result in genetic consequences, all else being equal. However, under our definitions, this is explicitly not an example of assortative mating. Thus, this scenario falls outside the scope of our statement about assortment on heritable traits.<br><br>*<u>Scenario 2 (Non-Heritable Sorting Factor):</u>*<br>If the sorting factor is environmentally correlated with the focal phenotype and not itself heritable, assortment on the sorting factor would not lead to increased genetic similarity. However, under our definitions, the sorting factor is the "assorted trait," not the focal phenotype. Therefore, if the focal phenotype is heritable but its sorting factor is not, the assortment is not on a heritable trait. Consequently, the statement remains valid, as it is restricted to scenarios where the assorted trait is heritable.<br><br>For scenario 2, one could imagine a scenario involving two heritable traits (X and Y) only correlated for environmental reasons. Assortment on X would produce a partner correlation in Y but without increasing genetic similarity for Y. However, there would still be increased genetic similarity for X. From the perspective of Y, the components of X associated with Y are not heritable (lines 168–169). Thus, this does not violate our statement about assortment on heritable traits leading to genetic similarity.

	In summary, both scenarios reflect processes that we argue cannot be described as assortative mating on heritable traits. There are numerous scenarios where partners can be phenotypically similar with no genetic similarity. However, if partner similarity is caused by assortative mating, and the assortment is on a heritable trait, then partners will be genetically similar for that trait (and associated traits that are genetically correlated with that trait).<br><br>In response to this (and other comments), we have revised and clarified our definitions of assortative mating and stratification elsewhere in the manuscript (e.g., see Table 1). For example, we have clarified the distinction between social stratification and social homogamy (i.e., assortative mating):<br><br>*Line 222:*<br>To avoid confusion, we suggest using the word *social stratification* (or just *stratification*) to refer to environmental correlations arising from partners sharing identical causes of variance, and reserve *social homogamy* for assortative mating on social characteristics, where covariance across partners arises from matching. This parallels the distinction between inbreeding (genetic variants identical by descent) and genetic homogamy (genetic variants with similar effects, but not necessarily identical).
**Minor**<br>- L 27: "Understanding indirect assortment is crucial for resolving the role of nature and nurture in education."<br>This sounds a bit vague for the general motivation of this research.	Thank you for this comment. We have attempted to revise the sentence to be less vague:<br><br>*Line 19 (Abstract):*<br>Understanding indirect assortment is crucial for disentangling genetic and environmental contributions to educational attainment and for improving models of intergenerational transmission.
- L 51 – typo: "progress have been hampered"<br>Should be "has"?	Thank you. We have corrected this typo.
- L 163f: "... Idiosyncratic homogamy for the degree to which non-shared environmental influences on the focal phenotype are associated with the sorting factor (e in Figure 1b)."<br>Could the authors provide a reasoning behind coining the non-shared environmental model "idiosyncratic"?	We settled on the term "idiosyncratic homogamy" after deliberating on alternatives, such as "unique homogamy" or "non-shared homogamy," which we consider to be less precise and too restricted to twin models. We wanted the terms to be applicable outside a twin-modelling framework, and idiosyncratic is a more general term than non-shared environmental factors (i.e., a person unfamiliar with twin models could still surmise what is meant by the word idiosyncratic).

	An alternative approach would have been to define "social homogamy" to include both family-shared and non-shared environmental factors. However, we chose to restrict "social homogamy" to family-shared environmental factors, as this aligns more with common usage in the field. This created the need for a distinct term to describe assortment on non-shared, idiosyncratic environmental factors. Rather than leave this concept undefined (which is also an option), we introduced "idiosyncratic homogamy" to fill the gap. We believe having a separate term for this can make it clearer that social homogamy typically means family-shared.<br><br>We have made a small revision, highlighting how the new definition fills a hole:<br><br>*Lines 180:*<br>We suggest defining social homogamy as the degree to which family-shared environmental influences on the focal phenotype are associated with the sorting factor ($\tilde{c}$ in **Figure 1b**). The specific operationalisation of family-shared environments may differ between studies (e.g., sibling-shared, twin-shared, parent-offspring shared, extended-family shared, et cetera), but we do not deem it worthwhile to define separate terms for all these contexts as their definition would simply follow how they are defined for the focal phenotype. This leaves undefined the degree to which non-shared environmental influences on the focal phenotype are associated with the sorting factor ($\tilde{e}$ in **Figure 1b**). We suggest using the term *idiosyncratic homogamy*. Other sources of variance (e.g., dominance effects, gene-environment correlations) can be added to the model following a similar logic, but we do not define separate terms for these.
- I've had been puzzle where the authors subsume structural/selection effects such as higher interaction density amongst highly educated due to University attendance which results in AM independent of preferences. This is discussed in detail but it seems subsumed under the social stratification model. The social stratification model, however, only focusses on C but such an effect can also emerge in E. I would prefer a general discussion of structural – also geographic effects – in a separate selection model. Selection versus preferences are discussed in respect to AM in detail in l 208f but I would prefer a separate model regarding this and the authors should note that disentangling	We thank the reviewer for raising this thoughtful point. We have revised our text to make our point more clearly.<br><br>We want to highlight that the distinction between stratification and assortment does not neatly align with the distinction between structural/selection reasons for partner similarity versus active choice and preferences. This is because many structural factors, such as university attendance, are themselves consequences of individual or family traits. As such, they make partner formation conditional on traits causing those structural factors and are therefore examples of assortative mating under our framework. Because we often hear structural factors such as university attendance forwarded as an alternative hypothesis to assortative mating, we want to explicitly counter this.<br><br>This reasoning is also why we have not included a separate selection model in the paper. Our framework does not aim to disentangle preferences from structural effects, as we believe this distinction does not matter for the aims of this paper.

structure/selection effects from preferences/homogamy is a larger discussion in the sociological literature for sure, see e. g.:<br>Skopek, J., Schulz, F., & Blossfeld, H. P. (2011). Who contacts whom? Educational homophily in online mate selection. European Sociological Review, 27(2), 180-195.	(We did test a model distinguishing social stratification from assortative mating and found no significant stratification effects, although this test is likely underpowered. We therefore refrain from drawing strong conclusions from it, and instead acknowledge that more research is needed [e.g., line 244])<br><br>That said, we recognize that stratification (i.e., exogenous structural factors) can, in principle, influence partner similarity without also being shared by siblings (i.e., emerging in E rather than C). However, we deem this unlikely. For this to be the case, the structural factor must somehow be exogenous to the traits in question, shared across partners, and not shared by siblings. In most cases, we believe structural factors associated with E would fall under the definition of assortative mating, or convergence.<br><br>We have made the following changes in response to your comment:<br><br>*Lines 234:*<br>This distinction can become muddled when the stratification itself is influenced by educational attainment or associated factors. Educationally segregated mating markets does not automatically imply social stratification unless these structural factors can be considered completely exogenous to the relevant traits. For example, if partner similarity in education could be wholly attributed to many would-be partners meeting at a university, then partner formation would be conditional on traits leading to university attendance, making this an example of assortative mating. Assortative mating does not by itself imply deliberate choice on behalf of the partners. Another example is non-random migration, where individuals move to areas because of their educational attainment or related factors rather than their educational attainment being decided by where they live[37,38]. If partners mate within the same area, but the area was decided by factors related to their educational attainment, then this would constitute assortative mating per the definition above. More research is needed on distinguishing stratification and homogamy.<br><br>*Line 603 (Final paragraph):*<br>Numerous questions remain. […] How relevant are preferences and active choice compared with educationally segregated mating markets[43]? […]<br><br>(Ref 43 is to Skopek et al 2011)

<table>
<tr>
<td>- I think it would be great if “indirect assortative mating” is defined in Table 1.</td>
<td>We have added this as a definition. We have defined it by exclusion (i.e., assortative mating that is not direct). We have also revised the definition of direct assortment so that the two definitions are contrastable.

<u>Table 1:</u>
Direct assortment:
Partner similarity arising due to assortative mating, where the focal phenotype and the sorting factor are identical. In other words, situations where the degree of genetic, social, and idiosyncratic homogamy equals the relative importance of genetic, social, and idiosyncratic influences on the focal phenotype.

Indirect assortment:
Partner similarity arising due to assortative mating, where direct assortment is an insufficient explanation for partner similarity. In other words, situations where the focal phenotype and the sorting factor are not identical.

Note that any of the non-assortment explanations automatically disqualifies direct assortment. For example, people cannot match on the phenotype that is already influenced by convergence. We have added a sentence about this:

<u>Line 207:</u>
Partner similarity need not result from assortment. Because we have provided new definitions for terms related to assortative mating, it will be beneficial to also describe other causes of partner similarity, and how they relate to assortment. <u>Note that any of the non-assortment explanations for partner similarity automatically disqualifies direct assortment as a sufficient explanation.</u> For example, partners can become more similar over time, [...].</td>
</tr>
<tr>
<td>- When the authors discuss the results in e. g. l 246f: “When accounting for this in intergenerational models, we found that 62% of the parent-offspring correlation could be explained by passive genetic transmission, whereas the remaining was explained by direct (15%) and passive (23%) environmental transmission.”, it might help the reader to mention the bias in the models not considering indirect AM explicitly since these models are the innovation.</td>
<td>We have revised the introduction accordingly:

<u>Line 280:</u>
When accounting for this in intergenerational models, we found that 62% of the parent-offspring correlation could be explained by passive genetic transmission, whereas the remaining was explained by direct (15%) and passive (23%) environmental transmission. <u>Models where we assumed direct assortment fitted poorly and gave results that suggested genetic transmission accounted for almost the entire parent-offspring correlation</u>.

(We do not give percentages, because that does not quite work when some of the components are negative).</td>
</tr>
</table>

- I got confused with the numbers of samples. The authors state there are: "1,529,144 individuals in 209,792 extended families", but then mention "2,407 244 monozygotic twins, 3,330 dizygotic twins, and 204,055 full siblings" as in 209,792 individuals. Which number is correct for individuals and families?	We apologise for unclear writing, and we have revised to make it clearer that the second set of numbers refers to the extended families of *pairs* of twins and siblings. The full context was that "we apply these models [...] using the extended families of 2,407 monozygotic twins, 3,330 dizygotic twins, and 204,055 full siblings". They add up to the 209,792-figure mentioned in the first set of numbers.<br><br>*Line 277*<br>[...] using the extended families of 2,407 pairs of monozygotic twins, 3,330 pairs of dizygotic twins, and 204,055 pairs of full siblings [...]"
- The authors state in l 254 that participants are born "...1975 and 1995 (and thereby old enough to have obtained education themselves)." It is fine that they are old enough that they had the opportunity to obtained education but to avoid right censoring they all should be beyond the observation window that allows still be in education - if that exists. However, it is realistic to still be in education when 29 years old nowadays. How did the authors deal with individuals still in education – e. g. a PhD.	We used the highest educational attainment recorded by age 30 for all individuals, even those who were considerably older at the time of observation. For individuals who completed a PhD after age 30 (e.g., at age 31 or beyond), they were classified as having a master's degree (or whatever highest education they had completed at age 30). This approach should ensure that younger individuals do not have artificially low education, and thus alleviate any problems caused by right censoring. We did not check whether individuals were still in education at age 30 as this was not readily available in the data file we used.<br><br>The three youngest cohorts in our sample (out of 20 cohorts in the offspring generation) did not reach the age of thirty by the time our data finishes (age 27 for the youngest cohort), so for them, we had to use the highest attained education up to that point. While it is possible that a minority of these would go on to complete more education in that time window, we do not believe this will have a large effect on our results. Furthermore, this only applies to the offspring generation in the iAM-COTS model. We chose to include them because they enabled us to identify more twins and twins-in-law in the parent generation, which are required to attain enough statistical power.<br><br>We have made some small revisions to make our approach clearer for the reader:<br><br>*Line 292 (Results):*<br>Finally, we linked the data to administrative education registers with information on educational attainment completed by age 30.

	Line 647 (Methods): For individuals born between 1992 and 1995 (who had not reached the age of 30 by the end of 2021), we used the latest available entry.
- In l 329 testing for measurement error, it says: "This resulted in significantly worse 330 fit than the full model" I was wondering why the authors term the model without measurement error "full model".	We have revised to make it clearer what this model is testing. The model in question is not testing for measurement error per se, but whether measurement error alone is sufficient to explain why direct assortment appears to be an insufficient explanation. That is, if the phenotype in question was undergoing direct assortment but was observed with measurement error, then this should look like a special kind of indirect assortment, which this model tests.<br><br>(Some of the changes in this paragraph are in response to Reviewer #2's comments)<br><br>*Line 368:*<br>A special kind of indirect assortment is when the phenotype is assorted upon directly but is observed with random measurement error[39]. Because partners do not assort on measurement error (which is modelled as part of non-shared environmental influences on the observed focal phenotype), idiosyncratic homogamy will be less important than implied under direct assortment (with genetic and social homogamy becoming correspondingly more important). Hence, the focal phenotype and sorting factor should have identical variance decompositions, except for non-shared environmental influences, which should be less important for the sorting factor. To test whether direct assortment with measurement error is a sufficient explanation, the model can be adapted by constraining $\tilde{a} = a$, $\tilde{t} = t$, and $\tilde{c} = c$ and freely estimating $\tilde{e}$. Compared with the full model, this removes three parameters and one constraint, giving two fewer degrees of freedom. Note that this leads to different variance for the sorting factor and the focal phenotype, meaning that a and $\tilde{a}$ may still be different after standardization (i.e., after rescaling to unit variance). This resulted in significantly worse fit than the full model (Δ -2LL = 463.9, $\Delta df = 2$, $p < .001$).
- The authors nicely demonstrate the utility of their extended framework in twin studies. I would really like to see a more extensive conceptual discussion how this impacts molecular genetically informed research and how PGSs can be used more felixible in this context etc.	We agree, and we are currently working on implementing polygenic scores in a similar design. That work is promising, but too extensive to include here. The reason for this is, among other things, that the way in which polygenic scores and the underlying GWAS's are affected by assortative mating is more complicated than family designs (especially with gene-environment correlations in the mix). An extensive discussion of that would be incomplete without a thorough treatment of these consequences, and that would require space we do not have here. Seeing as we are already above the journal's word limit, we have added instead decided to mention the possibility in the final paragraph where we talk about future directions:

	Line 614:<br>Future work remains on integrating multivariate assortment and indirect assortment under a common framework, and on applying this framework to molecular genetic designs.
- Overall, I really like this study and would wish these authors would write a similar paper on pleiotropy.	We thank the reviewer for their thorough evaluation of our paper, and for their suggestion of future papers. We (and other research groups) are thinking a lot about pleiotropy and, relatedly, multivariate assortment. We hope to be able to bring more clarity to those problems in the future.

Reviewer #2's comments	Our response and relevant changes
review by CVDolan of<br>Understanding indirect assortative mating and its intergenerational consequences by Hans Fredrik Sunde, Espen Moen Eilertsen, Fartein Ask Torvik.<br>Addresses the process underlying assortatitve mating (a.m.). This is an important subject generally, and a highly relevant subject given the present strong interest in genetic influences on socially relevant phenotypes, notable educational attainment (i.e., the rise of sociogenomics), and the possibly implications for social inequality, which characterizes Western societies. Includes a useful discussion of the often muddy terminology, and a conceptually explicit outline of possible processes, with explicit definitions. That is very useful. Includes an excellent application to educational attainment age age 30 in an impressive Norwegian data set (about 210.000 families, about 1.530.000 individuals). The application involves two designs: the iAM-ACE and iam-COTS, where the latter is better, but comes greater data requirements. I have comments, given below. Textual (minor) comments are given below (separate header).<br>My general impression is that this is well-executed important work, and that this paper will become highly influential, given its comprehensive approach to a.m.	We thank the reviewer for their overall positive appraisement and their thorough and helpful comments. We address them point by point below.
**Comments**<br>My general comment is that a lot of information is presented in the suppl. But I think some details could be included in the main text (if feasible, given word number limitations of the journal). See below.	We have added some more details about the models in the main manuscript in response to the reviewer's comments below. However, we are already above the journal's recommended word limit, so we don't believe adding more is feasible.

This paper focuses largely on EA. Should this be reflected in the title? The process giving rise to assortative mating, presumably, depends on the phenotype under consideration. Would it be useful to be explicit in this regard?	For context, the title of the paper is *Understanding indirect assortative mating and its intergenerational consequences*.<br><br>Our paper has two aims: (1) to clarify the framework used to understand partner similarity regardless of trait, and (2) to apply it to educational attainment. While the specific mechanisms of assortative mating likely differ by trait, the broader framework we present is generalizable. We have opted to retain the title to avoid giving the impression that the paper is solely about educational attainment, as our theoretical contributions extend beyond this single trait. If our argument is unsatisfactory, we are open to revising the title based on the reviewer's or editor's preference.
The paper considers EA, and positive assortment. That could be stated, so that other statements do not raise the issue of the sign of the spousal correlation (e.g.,217 "Under assortative mating, the correlation between a parent and their offspring will in most cases be inflated," i.e., positive assortment?)	We now specify that we are talking about positive assortment in the section where we first define assortative mating:<br><br>*Line 59:*<br>Finally, assortative mating is typically positive. It can in principle be negative (often called disassortative mating), but negative correlations between partners are rare[1]. In this paper, we will assume assortment means positive assortment unless otherwise stated.<br><br>*Line 246:*<br>Under positive assortative mating, the correlation between a parent and their offspring will in most cases be inflated<br><br>(The consequences of disassortative mating follows the same equations as for assortative mating, so it only matters for the descriptions given in plain English).
138 Figure 1 and 278. The term "copath" features twice in the text. I think that it would be useful to present this (if not 138, then 278) with a little more explanation, as it is a technical terms. E.g., identify it explicitly as the correlation that arises from the assortment process (matching on the sorting variable) in so far as it is manifest in the phenotype of interest.	We have added a sentence explaining it, with a reference to Cloninger (1980). In the interest of preciseness, we have avoided using the term "correlation" or "covariance" to describe what the copath coefficient is, as this is strictly speaking only true for standardized variables:<br><br>*Line 314*<br>The assortment strength itself is indicated with the copath coefficient μ. A copath indicates similarity due to matching, such as assortative mating, and comes with special path tracing rules that allow covariance to be induced without affecting variance[21].

"Finally, by defining homogamy with respect to the177 focal phenotype, consequences of assortative mating can be derived without involving global statements178 about partner formation".<br>Does this mean: without having to identify the process of partner formation, beyond the role of the sorting factor and its relationship with the causes of the phenotype (i.e., sources of phenotypic variance)?	Yes. We have revised this to make it clearer:<br><br>*Line 199:*<br>Finally, by defining homogamy with respect to the focal phenotype, consequences of assortative mating can be derived without involving global statements about partner formation (e.g., whether assortment results from active choices or not).

<table>
<tr>
<td>“This can come about from some form of190 assortative mating as described above, where covariance is induced without affecting the variance” This is an important point which could be presented more forcefully in the preceding text (& Fig 1).</td>
<td>We agree that this is an important point which should be highlighted more clearly. We have changed the text several places to make this point more forcefully:

<u>Line 55:</u>
Assortative mating is when individuals with similar trait values mate more often than expected by chance. More specifically, it is when partner formation is partly conditional on individuals’ having similar trait values, resulting in covariance between partners’ traits. Because the actual trait values remain unchanged, the variance is also unchanged. A defining feature of assortative mating is therefore that it induces covariance without affecting variance.

<u>Line 222:</u>
To avoid confusion, we suggest using the word social stratification (or just stratification) to refer to environmental correlations arising from partners sharing identical causes of variance, and reserve social homogamy for assortative mating on social characteristics, where covariance across partners arises from matching. This parallels the distinction between inbreeding (genetic variants identical by descent) and genetic homogamy (genetic variants with similar effects, but not necessarily identical).

<u>We have also revised the definitions in Table 1:</u>
Assortative mating:
Partner similarity arising because partner formation is conditional on a trait or set of traits (the sorting factor) associated with the focal phenotype, resulting in covariance between partners phenotypes without affecting the variance.

[...]

Social stratification:
Partner similarity arising for reasons independently of partner formation, because partners share identical, environmental causes of variance.

Inbreeding:
Partner similarity arising for reasons independently of partner formation, because partners share identical, genetic causes of variance. (i.e., genetic similarity due to genetic variants being identical by decent)

<u>And the figure legend for Figure 1:</u>
The copath (the arrowless path denoted μ) denotes assortative mating on the two connected variables, which results in covariance without affecting variances.</td>
</tr>
</table>

174 more generally, where the degree of genetic, social, and idiosyncratic homogamy is equal to the relative173 importance of genetic, social, and idiosyncratic factors on the focal phenotype ($a = \tilde{a}$, $c = \tilde{c}$, and $e = \tilde{e}$).<br>Here we see equalities. But later on the term proportionality is used (see line 230). Proportionality is more general than equality. So should a proportionality model be used here, rather than equalites? I think I that the phenotypic variance is not constrained, but the factor S is standardized, implying a~^2 + c~^2 + e~^2 = 1 (or later in iAM-ACE; a~^2 + c~^2 + e~^2 + t~^2 = 1). But if this is so, the $a = \tilde{a}$, is not likely ever to hold.	This is a good point. We have changed line 230 (now line 262) to state equality. Under direct assortment, the sorting factor will be identical to the focal phenotype, and therefore have the same variance as the phenotype. As a result, the issue of standardized variance for the sorting factor does not apply.<br><br>*Line 262:*<br>Unless the degree of genetic, social, and individual homogamy happens to equal the relative importance of genetic, social, and individual factors on the focal phenotype, models assuming direct assortment may give biased and misleading results.<br><br>*We have also revised the definition of direct assortment in Table 1*<br>Partner similarity arising due to assortative mating, where the focal phenotype and the sorting factor are identical. In other words, situations where the degree of genetic, social, and idiosyncratic homogamy equals the relative importance of genetic, social, and idiosyncratic influences on the focal phenotype.<br><br>More broadly, we think it is helpful to distinguish between (1) conceptual definitions and (2) how these definitions are implemented in models. In this context, we are referring to conceptual definitions, whereas the issue of standardizing the variance of S pertains specifically to model identification. There are also other possible identification constraints, such as fixing $\tilde{e}$ to either 1 or e, or setting the sorting factor variance to equal the phenotypic variance. The conceptual definitions should be agnostic to these modelling decisions.
198 different mechanism compared to assortment on social characteristics31<br>See also<br>https://link.springer.com/article/10.1007/BF01065887	We thank the reviewer for this suggested reference. We have added it as a reference where we discuss how the term "social homogamy" has been used differently:<br><br>*Line 123:*<br>For example, social homogamy is often implied to mean assortment on cultural factors only[27,32]
For example,202if potential partners cared about the socioeconomic position of their would-be parents-in-law, then this would203 be assortative mating.<br>should be: "...this would be social homogamy"?	We have changed it as suggested:<br><br>*Line 228:*<br>For example, if potential partners cared about the socioeconomic position of their would-be parents-in-law, then this would be social homogamy.

222 "Likewise, if a parent directly influences their children's222 traits, the other parent is likely to exert a similar influence, thus inflating the parent-offspring correlation."<br>I do not understand the stipulation / specification here that the other parent exert a similar influence.	In our original formulation, we conflated parents' trait value, the effect of a trait, and its consequence (the product of trait value and effect). We have attempted to rewrite to make it clearer:<br><br>*Line 249:*<br>For example, if partners are genetically similar, then their offspring will tend to inherit alleles with similar consequences from both parents, thus inflating the genetic similarity between one parent and their offspring (and hence also phenotypic similarity)[12]. Likewise, if offspring trait values are directly influenced by their parents' trait values, and both parents tend to have similar trait values, then the correlation between one parent and their offspring will increase because of the effect of the other parent
Cohorts are from 1975 to 1995. The correlations (fig 2) express the straightforward phenotypic relations, not fixed covariates, I assume?	The listed correlations are the observed phenotypic correlations.
The presentation of the models in the results section is as per journal requirement? The models are not really results.	Good question. We feel they make more sense in the current order as the purpose of the paper is to present a new model.
Figure 2. Possible to include the 95CI numerically next to the correlation (fig 2)? E.g., (say) Siblings/Twins ... n=3300 | r=.44 (.41 - .47).	We have added confidence intervals as text to the figure as requested.
Figure 2. It would be helpful perhaps to add to the figure caption examples of the relationships, so that it clear e.g., Avuncular-inlaw.	We have improved the labels in the figure (e.g., *Siblings-in-law* instead of *inlaws*), and added descriptions for those relationships which may be unclear:<br><br>*Figure 2*<br>[...] Siblings-in-law = partner of sibling/twin, Co-Siblings-in-law = partner of siblings-in-law, Avuncular = Sibling/twin of parent, Avuncular-in-law = Partner of sibling/twin of parent.
292 "This would imply a genotypic correlation between partners of .26 (.22, .29)." It would be instructive to add the calculation leading to .26. I assume that that can be done in a single sentence.	We have added a short sentence about how this is calculated:<br><br>*Line 333:*<br>Multiplying the heritability (.38) with the estimated partner correlation (.68) gives an implied genotypic correlation between partners of .26 (.17, .35).

Also, it would be useful to report how the phenotypic spousal correlation is decomposed into the latent component (A + the others).	This is not possible in a straightforward manner because of all the cross-partner correlations across different factors. The phenotypic spousal correlation would be the sum of 4x4 = 16 components (a weighted sum of the A-A correlation, A-C correlation, A-T correlation, etc.,), and we do not think reporting this decomposition will be that informative.<br><br>See also Supplementary Equation 5 (which omits twin-shared environments):<br>$$\rho_{partner} = a^2(\mu\tilde{a}^2) + c^2(\mu\tilde{c}^2) + e^2(\mu\tilde{e}^2) + ac(2\tilde{a}\mu\tilde{c}) + ae(2\tilde{a}\mu\tilde{e}) + ce(2\tilde{c}\mu\tilde{e}) \quad (5)$$

319 The test of phenotypic (direct) assortment fails, as it to be explected given the previous results. The test is one of " equality" constraints. As mentioned, I assume a~^2 + c~^2 + e~^2 + t~^2 = 1 so 3 df is understandable. The exact way the equality (or proportionality) in implemented could be mentioned in the main text, with ref to the relevant suppl. Later on the model is relaxed to estimate e~^2 freely. Again how this is done could be mentioned in the main text, again one or two sentences.	We have revised to make how the tests were conducted more explicit. First, we now mention how we constrained the variance of the sorting factor in the main text (elsewhere, we have mentioned how this can be done in different ways, such as by assuming unit variance or by fixing the non-shared environmental path. Here we mention how we chose to implement it. However, all of these options should be equivalent):<br><br>*<u>Line 313</u>*<br>To make the model identified, we constrained the variance of the sorting factor to equal the phenotypic variance.<br><br>Second, we describe in more detail how direct assortment was implemented:<br>*<u>Line 362:</u>*<br>To assume direct assortment, the degree of genetic, social, and idiosyncratic homogamy must be constrained to equal their importance for the focal phenotype ($a = \tilde{a}$, $c = \tilde{c}$, $t = \tilde{t}$, and $e = \tilde{e}$), which makes the focal phenotype and the sorting factor identical. This removes four parameters and one constraint, giving three fewer degrees of freedom.<br><br>Similarly, we provide more detail for the second model.<br>*<u>Line 370</u>*<br>[...]. Because partners do not assort on measurement error (which is modelled as part of non-shared environmental influences on the observed focal phenotype), idiosyncratic homogamy will be less important than implied under direct assortment. Hence, the focal phenotype and sorting factor should have identical variance decompositions, except for non-shared environmental influences, which should be less important for the sorting factor. To test whether direct assortment with measurement error is a sufficient explanation, the model can be adapted by constraining $\tilde{a} = a$, $\tilde{t} = t$, and $\tilde{c} = c$ and freely estimating $\tilde{e}$. Compared with the full model, this removes three parameters and one constraint, giving two fewer degrees of freedom. Note that this leads to different variance for the sorting factor and the focal phenotype, meaning that a and $\tilde{a}$ may still be different after standardization (i.e., after rescaling to unit variance). This resulted in significantly worse fit than the full model (Δ -2LL = 463.9, $\Delta df = 2$, $p < .001$).<br><br>We also made a small change in the supplementary regarding this model, as we had been a bit inaccurate when it came to the use of proportionalities versus equalities. We previously wrote *proportional*, but we changed it to *equal*. We also added that, despite being equated, they will differ after standardization. (This is what led to the inaccurate statement: The standardized parameters will be proportional, but the raw parameters will be equal).<br><br>*<u>Page 10 in the Supplementary:</u>*

	Another sub-model is direct assortment with measurement error, where $\tilde{e}$ are freely estimated while all other paths to the sorting factor are constrained to equal the corresponding path leading to the focal phenotype ($\tilde{a} = a; \tilde{c} = c; \tilde{t} = t; \tilde{e} \neq e$). Note that this leads to different variances for the sorting factor and the focal phenotype, meaning that a and $\tilde{a}$ may still be different after standardization (i.e., after rescaling to unit variance), but $\tilde{a}$, $\tilde{c}$, and $\tilde{t}$ will be inflated to the same degree.
338. The term "gene-environment correlation" is general. This could be expressed in terms of the actaul latent variables in the model, which would be more precise: I assume it is A – C covariance.	We agree that we should be more precise when describing how gene-environment correlations are modelled and have therefore reworded to make it clear that it is modelled as A-C covariance:<br><br>*Line 401:*<br>The COTS model can incorporate gene-environment correlations in the parent generation by including a covariance term, ω, between A_1 and C_1, where ω is defined to equal the equivalent correlation in the offspring generation.<br><br>*Figure legend for Figure 5*<br>[...] The parent generation is equivalent to the iAM-ACE model in Figure 2 (the only difference being the inclusion of a correlation between additive genetic effects (A_1) and sibling-shared environmental effects (C_1), denoted ω). The gene-environment correlation in the parent generation is constrained to equal that in the offspring generation: $\omega = Corr(A'_1 + A_2, F + C_2)$. [...]<br><br>We have also changed Figure 4 and Figure 7 to say V_{rAC} instead of V_{rGE}, as well as positioned the relevant column in between V_A and V_C.

344 “Because children of monozygotic twins will be as related to their parent as their parent’s twin, children of 344 twins can be used to decompose the parent-offspring correlation into parts attributable to genetic 345 transmission and various forms of environmental transmission”. Please state the “forms of environmental transmission” with meaning in natural language.<br><br>Consider: 489 etc. “ Our results 489 suggest that genetic similarity can account for much of this correlation (62%), but that a substantial part must 490 be ascribed to various forms of environmental transmission.” Important results, do statement of what the various forms are seem important to explicate.	We have revised to make it clearer what forms of environmental transmission we are referring to:<br><br>*Line 395:*<br>Because children of monozygotic twins will be as related to their parent as their parent’s twin, children of twins can be used to decompose the parent-offspring correlation into parts attributable to passive genetic transmission and two forms of environmental transmission operating within and outside the nuclear family (respectively called direct phenotypic transmission and passive environmental transmission, see **Supplementary Note 4**).<br><br>*Line 549:*<br>Our results suggest that genetic similarity can account for much of this correlation (62%), but that a substantial part must be ascribed to direct (15%) and passive (23%) environmental transmission.
**Minor textual. Not necessarily important: FYI.**<br>59 “Assortative mating occurs when individuals with similar traits mate more often than expected by chance.” “similar traits”. Ambiguous? I suppose that the phenotypic manifestation of assortative mating is the phenotypic correlation among partners. So assortative mating implies a non-zero phenotypic correlation among partners. “Similar traits” is strange, as everyone has these traits. Assortment is the similarity, spousal / partner correlation traits of interest.	Thank you for pointing out this inaccuracy. We have changed the wording to “trait values” rather than “traits”:<br><br>*Line 55:*<br>Assortative mating is when individuals with similar trait values mate more often than expected by chance.

63 "Assortative mating has important consequences for intergenerational transmission and will often bias63 estimates unless accounted for properly11,13,19." At this point is it not clear: estimates of what? Perhaps add…. often bias estimates of, for instance X, Y, Z, unless accounted …	We have rephrased to make it clear that, when it comes to intergenerational transmission, the bias is typically in the decomposition of genetic versus environmental effects. Here, we are writing in general terms, so we are on purpose being somewhat vague about whether we are referring to variance decomposition (A vs. C) or the decomposition of the parent-offspring correlation (the sentence applies equally to both).<br><br>*Line 69:*<br>[…] This can bias estimates of genetic and environmental effects unless accounted for properly[13,15].
75 "Numerous lines of evidence suggest that direct assortment is an inadequate explanation for partner similarity in educational attainment, and that partners must instead be more highly correlated on an associated variable."<br>The comparative: more highly, more highly than what? "… and that the phenotypic correlation among partners must be based on one or more associated variables." It seems too restrictive to assume "an associated variable", as the assortment may be based on two or more variables.	We have revised the sentence to reflect that it can be more than one variable, and to make it clearer that it is more similar than in educational attainment:<br><br>*Line 86:*<br>Numerous lines of evidence suggest that direct assortment is an inadequate explanation for partner similarity in educational attainment, and that partners must instead be more similar on associated variables.
78 "Robinson, et al. 6 found that education-associated genetic similarity between partners in the UK Biobank78 implied a correlation between partners in the assorted variable of .65, much higher than observed the79 phenotypic correlation for educational attainment at .41."<br>I assume: … implied an EA attainment correlation between the partners of .65". The variation is in terms: "education-associated" vs "eductional attainment". So the education-associated variable is EA?	We have revised to make it clearer. (On a side note, what this number (.65) refers to is unclearly written in the original paper, leading some (e.g., Clark 2023) to interpret it as the genotypic correlation between partners. However, it is clear from Robinson's surrounding explanation that it refers to the implied phenotypic correlation).<br><br>*Line 89:*<br>Robinson, et al[6.] found that education-associated genetic similarity between partners in the UK Biobank implied a phenotypic correlation of .65, much higher than the observed phenotypic correlation at .41.

89 "For example, Kemper, et al. 23 attempted to infer the89 phenotypic correlation between spouses' educational attainment by investigating phenotypic correlations90 between distant relatives in the UK Biobank."<br>"attempted" is ambiguous. "... Kemper, et al. inferred the ....?	We have revised accordingly:<br><br>*Line 100:*<br>For example, Kemper, et al. [23] inferred the phenotypic correlation between spouses' educational attainment by investigating phenotypic correlations between distant relatives in the UK Biobank.
93 "Similarly, Clark investigated correlations in social class among distant relatives in another large, English data set and found that genetic similarity alone could account for most of the familial resemblance if it were the case that partners correlated .79 on a highly heritable unobserved factor.<br>I lost the connect here with direct phenotypic assortment. I understand that the genetic similiarity is inconsistent with direct phenotypic assortment. But here a "highly heritable unobserved factor" is introduced. This factor is the direct assortment variable? But here the phenotype is social class. See also 95 ff: "The unobservable trait associated with EA".	We have revised to make these sentences clearer, and more consistent with previous sentences (unobserved factor is introduced in the previous paragraph):<br><br>*Line 103:*<br>Similarly, Clark [4] investigated correlations in social class (a phenotype closely associated with educational attainment) among distant relatives in another large, English data set and found that genetic similarity alone could account the familial resemblance if it were the case that partners correlated .79 on an unobserved factor associated with social class, and this factor was highly heritable. On the other hand, Collado, et al. [3] investigated educational attainment among distant family members in Sweden by chain-linking affine kinships (i.e., in-laws) and, while also finding that partners must be highly correlated (.75) on an unobserved factor associated with educational attainment, they ascribed this largely to assortment on non-genetic factors.

220 "For example, if partners are genetically similar, then offspring will tend to inherit genetic220 variants with similar effects from both parents, thus inflating the genetic similarity between a parent and their221 offspring (and hence also phenotypic similarity)12.<br>I find the additional "with similar effects" hard to follow. The effect of the genetic variant is given (ruling out age x gene interaction).	We agree we can be clearer on the distinction between trait, trait value, and effect. In this example, we must be careful: The key here is that offspring will not inherit the same variants per se (i.e., at the same loci), but variants at other loci with similar… well… effects. We have changed the wording to say "alleles with similar consequences" to make it clearer that we are talking about different genetic variants, and we are talking about their consequence (product of trait value and effect) rather than their effect per se:<br><br>*Line 249:*<br>For example, if partners are genetically similar, then their offspring will tend to inherit alleles with similar consequences from both parents, thus inflating the genetic similarity between one parent and their offspring (and hence also phenotypic similarity)
226 Models assuming direct assortment, such as most extended twin models with partners, implicitly226 assume that all pathways are inflated to the same degree, proportional to the phenotypic correlation between227 partners. Perhap mention what pathways you are referring to. I assume that this related to line 174.	We have revised to make it clearer that we are talking about intergenerational pathways:<br><br>*Line 257:*<br>Models assuming direct assortment, such as most extended twin models with partners, implicitly assume that all intergenerational pathways (e.g., direct transmission, passive genetic transmission, etc.,) are inflated to the same degree, proportional to the phenotypic correlation between partners.
233 There may be numerous ways to estimate the induced correlations in causes without assuming direct234 assortative mating.<br>"may be" is vague: There are numerous ways…	We have revised the sentence:<br><br>*Line 267:*<br>Here, we present a method to estimate the induced correlations in causes without assuming direct assortative mating.

Reviewer #3's comments	Our response and relevant changes
The paper by Sunde et al. "Understanding indirect assortative mating and its 7 intergenerational consequences" has many important contributions and insights into the phenomenon of assortative mating (in human) populations. The ambition of the paper is to develop a new framework, define key terms and apply the a new model to data on educational attainment from Norway.<br>As indicated, the paper has valuable elements, but I could not help that the claims might be too broad, that the treatment is not as fully comprehensive as suggested and that too much ignored from earlier work by others.	We thank the reviewer for their thorough and helpful comments, which we address below.
For example: line 43/44: "Mating partners tend to have similar educational attainment": this statement is based on a single population, i.e. UKB which not only has an overrepresentation of more highly educated (female) participants but also comes from a country with a class system that is more culturally ingrained than in many other countries. The difference between the UK and Sweden for example (lines 88-102) could also explain the differences in the results as noted? The authors acknowledge that cultural differences may exist (between UK and Norway -see discussion- but deem these unlikely as an explanation for differences in results?).	This statement is *not* based on a single population. We cite Horwitz et al (2023), a meta-analysis of partner similarities in various traits (which was indeed supplemented with analysis of traits in the UKB, although this is only part of the paper). Their estimate of partner similarity of educational attainment is based on 86 estimates from 42 culturally diverse studies (with samples from e.g., China, Latin America, the EU, the US, India, South Korea, and indeed Norway). While there is, as expected, some heterogeneity between studies and cultures, we think this reference is more than sufficient to support the general claim that "mating partners tend to have similar educational attainment.". In their forest plot (Figure S1T on page 23 of their supplement), we see that all included correlations are both significant and reasonably large (In both the UK and the Nordics, it is typically reported at between .40 and .50).<br><br>Cultural differences in mating behaviour undoubtably exist and will affect the answer to the research questions raised in this paper. However, we find it unlikely to be the primary explanation for the discrepant results in the literature. There are three reasons for this: First, as the reviewer writes, the class system is probably more culturally ingrained in the UK than in many other countries (cf. the Nordics, where many of the other studies are from). We would then perhaps expect a cultural explanation was more likely in the UK studies than in, say, Swedish studies. Instead, the studies from Sweden (Collado, Reynolds) tend to suggest a cultural explanation while UK studies (Clark, Kemper) tend to suggest a genetic explanation. Second, there are methodological

	differences between the studies that can partly explain their results (we discuss this on *lines 127-134* and *lines 532-542*). This provides a simpler explanation for the discrepancy that must be accounted for before invoking cultural differences. Third, our results are consistent with both "sides" of the debate (both more genetic and social homogamy than expected under primary assortment), which partly obviates the need to explain the discrepant findings. As such, we view cultural differences as an important follow up question (we write this on *line 609*), but unlikely to be the primary explanation for the extant discrepant results in the literature.
The sentence "Researchers broadly agree..": I am not sure that this is backed up by references 2-8.	We have revised the sentence to make it less strong. Rather than stating that researchers agree that it must be mating on other traits *rather than* EA, we now say "rather than, *or in addition to*", as this more closely align with the conclusions of some of the cited papers (e.g., Gonggrijp and Reynolds use a model where social homogamy is happening "on top of" primary phenotypic assortment, and as such do not conclude that the assorted trait is anything other than EA, per se. However, they do conclude that direct assortment alone cannot account for the partner correlation):<br><br>*Line 38:*<br>Researchers broadly agree that this results from assortative mating on associated traits rather than, or in addition to, education itself, as the partner resemblance in factors relevant for educational attainment is estimated to be higher than expected given resemblance in observed educational attainment[2-9].<br><br>As for whether "researchers broadly agree" on this statement: All seven studies have as one of their central claims that direct assortment alone is an insufficient explanation for partner similarity in educational attainment. We are not aware of any recent papers that have explicitly tested whether direct assortment is a sufficient explanation and concluded that it is. Hence, we deem this statement a fair description of the current opinion among relevant researchers.<br><br>We have added an eight reference (Phillips et al, 1988), which the reviewer asks us to cite below.
Maybe similar, the references 9-14 include books that are so broad (e.g. an intro textbook of behavior genetics) that is hard to see how a reader can understand why it is important	References 9-14 supported the claim that statistical models, in particular genetic models of intergenerational resemblance, are biased in the presence of unmodelled assortative mating. This included briefer papers that go into detail about how assortative mating (often among other things) can bias various estimators, as well as one longer introductory textbook to behaviour genetics.

correctly model assortment in order to understand social inequality.	We cited the textbook as a reading suggestion, not only to substantiate this claim. We have removed the reference to the book (it is not strictly needed). We also removed the reference to Fisher 1919 here (it is cited elsewhere in the paper). Despite being the seminal paper describing the genetic consequences of assortative mating, it does not describe modelling biases per se.<br><br>We also added a few more papers describing AM-related biases in various statistical models. The reader will have the option of pursuing those most relevant to their field.<br><br>*Reference list*<br>13 Coventry, W. L. & Keller, M. C. Estimating the extent of parameter bias in the classical twin design: a comparison of parameter estimates from extended twin-family and classical twin designs. *Twin Research and Human Genetics* **8**, 214-223 (2005). https://doi.org/10.1375/1832427054253121<br>14 Hartwig, F. P., Davies, N. M. & Davey Smith, G. Bias in Mendelian randomization due to assortative mating. *Genetic Epidemiology* **42**, 608-620 (2018). https://doi.org/10.1002/gepi.22138<br>15 Border, R. *et al.* Simple models of non-random mating and environmental transmission bias standard human genetics statistical methods. *bioRxiv* (2024). https://doi.org/10.1101/2024.10.16.618755
Line 59: assortative mating (AM) occurs …: maybe ‘describes would be better?	We have changed to "is".<br><br>*Line 55:*<br>Assortative mating is when individuals with similar trait values mate more often than expected by chance.
There are multiple consequences of assortative mating for traits influenced by genetics, including greater homozygosity for loci related to these traits. Over generations, this can amplify genetic similarities within subgroups of a population, even leading to reduced genetic diversity within subgroups, but in the population as a whole to an increase in genetic variation (see e.g. Falconer: Introduction to Quantitative Genetics). A focus on bias in parameter estimates (line 64) may be important,	We have revised the paragraph where we first mention genetic consequences of assortative mating. We now highlight many consequences (incl. homozygosity), and that the magnitude of these consequences depends on the genotypic correlation between partners. This motivates the distinction between direct and indirect assortment:<br><br>*Line 62:*<br>When mating is conditional on specific traits, the causes of these traits (and other associated factors) will become correlated across partners. For example, if the traits are associated with genetic differences, partners will tend to be genetically similar. Numerous studies have demonstrated genetic similarity between partners for traits like educational attainment[6,7,16-19]. Genetic similarity between partners will have numerous consequences in subsequent generations, including long-range linkage disequilibrium between trait-associated loci, increased genetic variance in the population, increased genetic similarity between relatives, and, for traits influenced by few loci, increased homozygosity at trait-associated loci[17,20-25]. This can bias estimates of genetic and environmental effects unless accounted for properly[11,13]. The magnitude of these consequences (and hence the potential biases) is a function of the genotypic correlation between partners.

but does not seem a 'consequence of AM'? (see also the paragraph beginning at line 216).	Because this correlation is often unknown, it is typically inferred based on the questionable assumption that assortment takes place directly on the observed phenotype – henceforth *the focal phenotype* (**Figure 1a**). This is called *direct assortment*, or primary phenotypic assortment[26]. Under direct assortment, the expected genotypic correlation between partners is known, and the consequences and necessary adjustments are well understood (see **Supplementary Note 1**). However, matching on a phenotype will also lead to partner correlations on associated phenotypes[27]. For example, partners assorting on height will have correlated arm lengths as well[23]. These secondary partner correlations are said to result from *indirect assortment*, or secondary assortative mating[7]. [...]<br><br>Other than a brief mention in the above paragraph, we have not discussed homozygosity further in our manuscript. This is because it is only of practical importance for traits that are monogenic or influenced by only a few loci. (The description in Falconer's textbook (3e, p.21) only pertains to a single locus). For polygenic traits, the increase in homozygosity is negligible (we show why below). Because complex traits are typically *very* polygenic (ref *The Fourth Law of Behavioural Genetics*, Chabris 2015. https://doi.org/10.1177/0963721415580430), homozygosity is not an important consequence of assortative mating for such traits.<br><br>The relationship between polygenicity and homozygosity:<br>The effect of assortative mating on homozygosity are described several places. Here, we base our description on Wright (1921, https://doi.org/10.1093/genetics/6.2.144). At equilibrium, the proportion of heterozygotes at a given locus (p) is given by the following equation (assuming allele frequencies of 50% and equal effects of all loci for simplicity):<br><br>$$p = \frac{n(1-m)}{2n(1-m)+m}$$<br>We can use this to calculate the proportion of homozygotes (1-p) we can expect at equilibrium for different degrees of polygenicity (number of loci, denoted n) and different genotypic correlations between partners (denoted m). As we can see in the figure below, the increase in homozygosity quickly becomes negligible for traits that are even just modestly polygenic. For reference, GWAS of complex traits like height or educational attainment typically find thousands of relevant loci (12,111 and 3,952 for height and EA, respectively).

	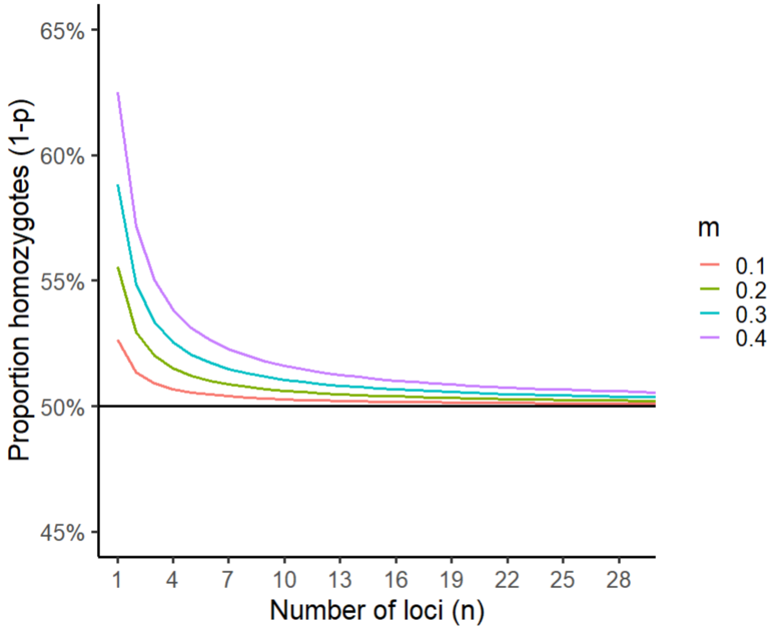<br><br>The reason for why the increase in homozygosity is negligible for polygenic traits is quite intuitive: The degree of homozygosity at a given locus depends on the partner correlation at that locus in the preceding generation. However, the partner correlation at a given locus is only a fraction of the genotypic correlation between partners: The genotypic partner correlation (m in the equation above) is the sum of all cross-partner correlations within and across loci (weighted by the loci's effects). The more loci, the smaller part a given locus plays in the correlation, until it becomes negligible. This is also why dominance effects on polygenic traits are practically unaffected by assortative mating (see response below).
Line 64/65: Most prior …. I am not sure this is true and would have expected a reference to e.g. Phillips et al. (DOI: 10.1007/BF01260935) who proposed to model multivariate assortment in order to test for the presence of an indirect marital correlation (in their case between education in one spouse and intelligence in the	We thank the reviewer for this thoughtful comment. The paragraph with this statement has been revised in response to other comments. The original sentence read: "Most prior attempts to account for it have assumed that the phenotype in question – henceforth *the focal phenotype* – is assorted upon directly (**Figure 1a**)". The equivalent, revised sentence now reads:<br><br>*Line 71:*

other), testing if the correlation is due only to the direct pairing on education and on intelligence and to the within-person correlation between the two traits.	The magnitude of these consequences (and hence the potential biases) is a function of the genotypic correlation between partners. Because this correlation is often unknown, it is typically inferred based on the questionable assumption that assortment takes place directly on the observed phenotype – henceforth *the focal phenotype* (**Figure 1a**).<br><br>While Phillips et al. (1988) offers an important contribution to understanding multivariate assortment, we believe it is not directly relevant to the specific point made in this sentence. (It does not attempt to account for assortment, direct or otherwise, but instead inform on assortment within and across traits). Incidentally, they also conclude that direct assortment on education is insufficient to explain the partner correlation in education: Their best-fitting model explains the partner correlation in education (.43) as **the sum of** (1) assortment on education (.40) **and** (2) assortment on intelligence (.11) weighted by the intelligence-education correlation in men (.45) and women (.41). (They don't find evidence of cross-trait assortment, but that is not relevant here). In other words, the partner correlation in education could not be entirely attributed to direct assortment on education: Indirect assortment (via intelligence) had to be included in their model. We have added Phillips as a reference when we state that researchers broadly agree that partner similarity in education cannot be fully ascribed to direct assortment on education (line 46, see Reviewer #3's second comment).
Introduction of models: why is genetics / genotype reduced to additive genetic influences? In a plea for rigorous terms, I would hope to see a treatment of the full genotype (A+D).	We have made a revision where we discuss the potential distinction between additive and genetic homogamy:<br><br>*Line 171:*<br>Using this framework, we suggest defining genetic homogamy as the degree to which the genetic influences on the focal phenotype are also associated with the sorting factor ($\tilde{a}$ in **Figure 1b**). For traits influenced by both additive and non-additive (e.g., dominant) genetic effects, it can be useful to distinguish additive and dominant genetic homogamy. However, even though dominant genetic homogamy will lead to a correlation between partner's dominance deviations, the intergenerational consequences of this are negligible for polygenic traits[18,24,26]. Dominance effects are also rare for complex traits[40]. Because of this, we henceforth only consider additive genetic homogamy<br><br>(One of the references [18] are to an earlier paper of ours, where we used simulations to examine the intergenerational consequences of AM on dominance effects. As theoretically predicted, there were none). We have also included a sentence highlighting how other sources of variance can be added to the model following a similar logic (i.e., separate effects on focal phenotype and sorting factor):<br><br>*Line 188:*

	Other sources of variance (e.g., epistasis effects, gene-environment correlations) can be added to the model following a similar logic, but we do not define separate terms for these.
Also, I struggle with the definition of family-shared environment: suppose partner 1 has a sib-environment with multiple brothers and sisters whereas their partner is from a single offspring family. They cannot assort on the shared environment? What happens to the variance induced by the shared environment?	We thank the reviewer for highlighting this potential source of confusion. In behavioural genetics, *shared environment* refers to environmental influences that contribute to sibling similarity, regardless of whether an individual actually has siblings. These influences—such as parental socioeconomic status, stable parenting practices, and neighbourhood characteristics—would be shared if siblings were present and exist even in the absence of siblings. Therefore, the concept applies equally to individuals from single-child families.<br><br>We have added the following sentence:<br><br>*Line 322:*<br>Note that sibling- and twin-shared environmental factors reflect influences that would be shared with siblings or twins, regardless of whether an individual actually has them.
Line 477 and further: the summary of the results of other studies does not seem entirely adequate (e.g. Gonggrijp et al. conclude "both social homogamy and phenotypic assortment are important processes in spouse selection in Finland and the Netherlands. In both countries, phenotypic assortment contributes to a greater degree to the similarity of spouses than social homogamy").	The model used by Reynolds and later Gonggrijp used a slightly different conception of social homogamy than presented here. In their models, partners are modelled as assorting on *both* the phenotype and social influences, independently of each other. The model in Collado's papers is similar, although the assumption that the two assortment processes are happening independently of each other is relaxed. These differences make their results not directly comparable to ours, which led to the slightly inaccurate summary in our original manuscript. We have revised to make the summary more accurate, while still making their results comparable to ours:<br><br>*Line 535:*<br>All three of these studies concluded that environmental partner similarity was higher than expected under direct assortment alone, and hence an important explanation for partner similarity in educational attainment. In this sense, our study can be said to replicate their results.

Response to Reviewer #3

Please let my observations not distract from the fact that there are many valuable points and results in this paper. I would ask the authors to adjust their phrasing.	Thank you. We have attempted to adjust our phrasing where needed.

Response to reviewers

Reviewer #1's comments	Our response and relevant changes
Thank you for the opportunity to read the revised version of the manuscript 'Understanding indirect assortative mating and its intergenerational consequences'. The reviewers have thoroughly addressed all my comments and I would like to see this paper as part of the literature. I have only one outstanding comment: We engaged in a discussion on whether the are at 30 is the end of the observation window for educational attainment, and it turned out that some of the folks are even only observed until they are 27 years old. I would like to see this as part of the discussion section as a limitation of the empirical study. Other than that, I would like to congratulate the authors for their great work.	We thank the reviewer for their careful and detailed assessment of our manuscript. The thorough engagement has been extremely helpful in refining the paper. We have now added a note in the discussion section acknowledging the limitation regarding the observation window for educational attainment (although this applies only to the offspring generation, and not the partners):<br><br>*Line 579:*<br>Another limitation is that some individuals in the offspring generation were only observed until age 27, which may slightly underrepresent later educational attainment.

Reviewer #2's comments	Our response and relevant changes
Review by CVDolan of the revision of "Understanding indirect assortative mating and its intergenerational consequences"<br>bby Hans Fredrik Sunde1*, Espen Moen Eilertsen2, Fartein Ask Torvik1,2<br><br>The authors have adequately addressed my previous points. My subsequent comments concerns mainly textual edits. I am happy to leave it to the authors whether to act on these or not. Line numbers are including in the quoted text (identifying the location in the ms). I have a few substantive comments (indicated with the ***). I imagine that these can be addressed easily.<br><br>I consider this an excellent paper, truly "a steppingstone in ...the right direction". The fact that these models require huge sample sizes does not detract from the conceptual and theoretical analysis of the issue of assortment.	We sincerely appreciate the reviewer's thoughtful and detailed comments, as well as the generous assessment of our work. The careful attention to both conceptual and textual aspects has been especially helpful in refining the manuscript. Below, we address the remaining comments and have made revisions where appropriate.

Response to Reviewer #2

"Mating partners tend to have similar educational attainment1. Researchers broadly agree that this results from38 assortative mating on associated traits rather than, or in addition to, education itself, as … "<br><br>Interpunction? Suggestion:<br><br>Mating partners tend to have similar educational attainment1. Researchers broadly agree that this results from assortative mating on associated traits, in addition to possible assortment on education attainment itself, as …	We have revised the sentence:<br><br>*Line 39:*<br>Researchers broadly agree that this results from assortative mating not only on education itself, but also on associated traits, as partner resemblance in factors relevant for educational attainment is estimated to be higher than expected given resemblance in observed educational attainment
"More specifically, it is when partner formation is partly conditional on individuals' having similar trait56 values, resulting in covariance between partners' traits."<br><br>More specifically, it arises when …	We have revised the sentence:<br><br>*Line 55:*<br>Assortative mating arises when individuals with similar trait values mate more often than expected by chance
"on the questionable assumption that assortment takes place73 directly on the observed phenotype"<br><br>Arguably questionable in a study of educational attainment. But not generally questionable. Therefore: "on the strong assumption that …. "	We agree that it is better to write *strong* than *questionable*, and have revised the sentence:<br><br>Line 71:<br>Because this correlation is often unknown, it is typically inferred based on the strong assumption that assortment takes place directly on the observed phenotype – henceforth *the focal phenotype*.

"Under direct assortment, the expected genotypic correlation76 Understanding indirect assortative mating [...] 2 between partners is known, and"<br><br>"is known"? Does this not require additional information? Can be estimated? If I say the mating is due to direct assortment and the spousal phenotypic correlation is .3, you do not know the genotypic correlation (as it depends on the other parameters in the model, plus all the assumptions about "interplay").	We agree that our original wording could be misleading, as it is of course only known as a function of other parameters in the model. (i.e., it can be easily calculated *if* you know the phenotypic correlation *and* the phenotype-genotype correlation). We have reworded the sentence to make it clearer that it must still be calculated based on other "known" quantities:<br><br>*Line 74:*<br>Under direct assortment, the expected genotypic correlation between partners can easily be calculated, and the consequences and necessary adjustments are well understood (see **Supplementary Note 1**).
"However,78 matching on a phenotype will also lead to partner correlations on associated phenotypes28."<br><br>Here you are still referring to direct assortment. If so please state. "However, direct assortmnet on a phenotype..."	Yes, we have reworded the sentence to make this explicit:<br><br>*Line 75:*<br>However, direct assortment on one phenotype will also lead to partner correlations on associated phenotypes
"that partners must instead be more highly correlated similar on an associated87<br>variables."<br><br>ambiguous comparative: more similar than what? The message is that the assortment is on traits related to EA, I think.	We have revised to disambiguate the comparative:<br><br>*Line 83:*<br>Numerous lines of evidence suggest that direct assortment is an inadequate explanation for partner similarity in educational attainment, and that partners must instead be more similar on variables associated with education.
106 "Hindrances for understanding of indirect assortative mating"<br>Hindrances to	The subheading now reads:<br><br>*Line 110:*<br>**Hindrances to understanding of indirect assortative mating**

"For example, social homogamy is often implied to mean122 assortment on cultural factors only27,32."<br><br>It would perhaps be useful to explicate "cultural factors". Ambiguous term ? What are these? I assume "environmental" (given the text later on: "model environmental similarity between family members, and thereby precluded a cultural explanation"). Anyway: "cultural factors" is an ambiguous term. This is dealt with later on. It might help the reader to state that fact.	We initially struggled with the structure of the manuscript, especially with where to place the critical discussion of the term "social homogamy". We have revised this sentence to use the less ambiguous (but admittedly also more vague) term "environmental":<br><br>*Line 117:*<br>For example, social homogamy is often implied to mean assortment on environmental factors only
*** Fig 1: I like the clear representation of competing models. We see that A and C are uncorrelated in model 1b (the model of interest here) and we see that A and C are uncorrelated in 1e. I wonder whether it is desirable at this point to state explicitly this aspect of these model, and whether the presence of AC covariance has any bearing. This is specifically of interest in the case of AE, which presumably is subject to AC cov. Intergenerational equilibrium may hold with respect to the genetic nature process. I suppose that this is dealt with in the second model (see line 391). Maybe useful to alert the reader to this here?	We have revised the figure caption to highlight that A-C covariance and other forms of gene-environment correlations have been omitted from the models for simplicity's sake and can be added should it be relevant for a given phenotype. (We hope it is clear that the general framework where the focal phenotype and its sorting factor are treated separately does not rely on assumptions about no A-C covariance. Instead, it is omitted simply to ease the explanation). We have also revised the paragraph where we define genetic and social homogamy to highlight that A-C covariance can intertwine the consequences of genetic and social homogamy.<br><br>*We have added the following to the figure legend for Figure 1 (Line 146):*<br>For simplicity's sake, we assume no correlations between genetic and environmental influences, but this can be added to the models when relevant (see also Figure 5).<br><br>*Line 189:*<br>Other sources of variance can be added to the model following a similar logic, but we do not define separate terms for these. Variance attributable to correlations between social and genetic influences will intertwine the consequences of social and genetic homogamy (e.g., social homogamy can have genetic consequences), but we do not consider this its own form of homogamy.<br><br>Relatedly, we also made a small change to the section on genetic homogamy:<br><br>*Line 173:*<br>For traits influenced by both additive and non-additive (e.g., dominant) genetic effects, it can be useful to distinguish narrow-sense sense (additive only) and broad-sense (additive and non-additive) genetic homogamy. We urge readers to be explicit if they model varied forms of genetic homogamy. In this paper, we will only consider narrow-sense genetic homogamy.

”Only the relative importance of $\tilde{a}$, $\tilde{c}$, $\tilde{t}$, and $\tilde{e}$ can be estimated, meaning the variance of the sorting factor must be348 constrained.”<br><br>Indeed this was mentioned earlier “To make the model identified, we constrained the variance of the sorting factor313 to equal the phenotypic variance.”<br>So perhaps this could be mentioned explicitly in figure caption (248).	While we appreciate this comment, we have eventually decided to leave it unchanged (but see next comment and our response). Our logic is that the figure and figure caption present a proposed model that other researchers may want to consider. In this context, it is only relevant that the variance needs somehow to be constrained. This can be done in several ways, for example by non-linear constraint or by fixing one of the paths. We therefore were intentionally a bit vague in the figure caption. On the other hand, for our empirical results, we need to describe how *we* decided to constrain the variance.

*** “To assume direct assortment, the degree of genetic,362 social, and individual assortment idiosyncratic homogamy must be constrained to equal their importance for363 the focal phenotype ($a = \tilde{a}$, $c = \tilde{c}$, $t = \tilde{t}$, and $e = \tilde{e}$), which makes the focal phenotype and the sorting364 factor identical. This removes four parameters and one constraint, giving three fewer degrees of freedom. We365<br>find that constraining the model to direct assortment results in substantially worse fit (Δ -2LL = 8590.0, Δdf366 = 3, p < .001, Supplementary Figure 12)”.<br><br>We read that that var(S) = var(focalPh), i.e., an identification constraint. The parameters $\tilde{a}$, $\tilde{c}$, $\tilde{t}$, and $\tilde{e}$, are free parameters initially. The equality constraints ($a = \tilde{a}$, $c = \tilde{c}$, $t = \tilde{t}$, and $e = \tilde{e}$) implies + 4 df. So I assume that once these constraints are in place, the constraint var(S) = var(focalPh) is no longer necessary, so that implies -1 df resulting in +3 df? It would be useful to state explicitly “This removes four parameters and allows us to relax the constraint that the variance of S equals the variance of the focal phenotype”.<br><br>It this is correct, are the models actually nested (justifying the use of the LRT)? I understand that<br>model A: ($a = \tilde{a}$, $c = \tilde{c}$, $t = \tilde{t}$, and $e = \tilde{e}$) and var(Ph) = var(S)<br>is nested under<br>model B: (a != $\tilde{a}$, c != $\tilde{c}$, t != $\tilde{t}$, and e != $\tilde{e}$) and var(Ph) = var(S)<br><br>But is<br>model A: ($a = \tilde{a}$, $c = \tilde{c}$, $t = \tilde{t}$, and $e = \tilde{e}$) and var(Ph) =	We thank the reviewer for this interesting and thought-provoking comment. It is correct that, under direct assortment (when $a = \tilde{a}, c = \tilde{c}, t = \tilde{t}$, and $e = \tilde{e}$), the equality constraint $Var(S) = Var(P)$ is no longer necessary. Not only is it no longer necessary, but even if it was included in the model, it would no longer contribute a degree of freedom because $Var(S) = Var(P)$ is already implied by the equality constraints on the other parameters. In other words, models A and C are identical and have the same degrees of freedom.<br><br>Now to the more substantive question of whether the models are nested or not: This is a great question. Below, we explain why we do not think this is an issue, but as other readers may share similar concerns, we have re-estimated the models using a parameterization that leaves no room for doubt whether the models are nested. Instead of using an explicit variance constraint, we accomplish this by fixing one of the paths to the sorting factor and rescaling the paths afterwards. To make sure all the sub-models we want to test (direct assortment $[a = \tilde{a}, c = \tilde{c}, t = \tilde{t}$, and $e = \tilde{e}]$ and direct assortment with measurement error $[a = \tilde{a}, c = \tilde{c}, t = \tilde{t}$, and $e \neq \tilde{e}]$)) are definitely nested under the full model, we fix $\tilde{a}$ to a. We fix $\tilde{a}$ instead of $\tilde{e}$ because $\tilde{e}$ needs to be freely estimated in the second sub-model, so it needs to be freely estimated in the full model too. (Note that $\tilde{a}$ to a can still differ after they are standardized, meaning we are not assuming that genetic homogamy and heritability are proportional or equal). Now, the comparison between the full model ($[a = \tilde{a}, c \neq \tilde{c}, t \neq \tilde{t}$, and $e \neq \tilde{e}]$) and the sub-model with direct assortment ($[a = \tilde{a}, c = \tilde{c}, t = \tilde{t}$, and $e = \tilde{e}]$) is simply a comparison where the $\tilde{c}$, $\tilde{t}$, and $\tilde{e}$ parameters are or are not freely estimated.<br><br>We have updated all figures, tables, and result files with the results from the re-estimation, and checked all numbers presented in text. They are all the same barring a few changes at inconsequential decimal places and some numbers that are rounded differently. We have also updated the text in the relevant places:<br><br>*Line 306:*<br>To make the model identified, one needs to constraint the variance of the sorting factor by fixing one parameter. We chose to fix $\tilde{a}$ to a. Note that this leads to different variance for the sorting factor and the focal phenotype, meaning that a and $\tilde{a}$ may still be different after standardization (i.e., after rescaling to unit variance).<br><br>*Line 357:*

var(S)<br>nested under<br>model C: ($a = \tilde{a}$, $c = \tilde{c}$, $t = \tilde{t}$, and $e = \tilde{e}$) and var(Ph)<br>!= var(S)<br>?<br><br>If is presumably possible to step down from B to A (4 df) to C (1 df). Here the substantive meaning of the comparison A vs C requires explication. see also line 381 and lines 432-433.	To assume direct assortment, the degree of genetic, social, and idiosyncratic homogamy must be constrained to equal their importance for the focal phenotype ($a = \tilde{a}$, $c = \tilde{c}$, $t = \tilde{t}$, and $e = \tilde{e}$), which makes the focal phenotype and the sorting factor identical. Since $\tilde{a}$ was already fixed, this gives three fewer degrees of freedom.<br><br>*Line 367*<br>To test whether direct assortment with measurement error is a sufficient explanation, the model can be adapted by constraining $\tilde{a} = a$, $\tilde{t} = t$, and $\tilde{c} = c$ while still freely estimating $\tilde{e}$. Compared with the full model, this removes two degrees of freedom.<br><br>*Line 660*<br>Only the relative importance of $\tilde{a}$, $\tilde{c}$, $\tilde{t}$, and $\tilde{e}$ can be estimated, meaning the variance of the sorting factor must be constrained. We accomplished this by fixing $\tilde{a}$ to equal a and rescaling the parameters afterwards.<br><br>---<br><br>**As for whether it truly matters:** Our thinking is that it, for the full model, it should be arbitrary how you constrain the variance of S, and that the various parameterizations that accomplishes this are equivalent. Therefore, a model with an explicit variance constraint ($Var(S) = Var(P)$, Model 1) should be equivalent to a model where one of the paths is fixed and all paths rescaled afterwards (e.g., $\tilde{e} = 1$, Model 2). Furthermore, it should be arbitrary which path is fixed (e.g., $\tilde{a} = 1$, Model 3), and what it is fixed to (e.g., $\tilde{a} = a$, Model 4). Indeed, these four approaches yield the same likelihood down to a tolerance of $\Delta LL < .001$ (see screenshot below), meaning any likelihood ratio test should also give the same results down to a similar tolerance. Consequently, we believe that if a model can be shown to be nested under any of these models, they can be considered nested under all of them. As described above, a model assuming direct assortment is definitely nested under Model 2. We therefore believe it can be considered nested under Model 1 as well. (Although, now that we have reparametrized the model used for the empirical results, this does not really matter anymore).

```
>
> models <- list(fit_M1,fit_M2,fit_M3,fit_M4)
> mxCompare(models)
                             base comparison ep minus2LL     df     AIC diffLL diffdf  p
1   Model 1 (VarS constrained)       <NA> 10  2230654 836260 2230674     NA     NA NA
2 Model 2 (e_tilde fixed to 1)       <NA>  9  2230654 836260 2230672     NA     NA NA
3 Model 3 (a_tilde fixed to 1)       <NA>  9  2230654 836260 2230672     NA     NA NA
4 Model 4 (a_tilde fixed to a)       <NA>  9  2230654 836260 2230672     NA     NA NA
>
> # Find largest likelihood difference:
> max(unlist(lapply(models, function(x) x$output$fit))) - min(unlist(lapply(models, func
[1] 0.0009103026
> |
```

"we found that 62% of the479 parent-offspring correlation could be explained by passive genetic transmission, whereas the remaining was480 explained by direct (15%) and passive (23%) environmental transmission"<br>"....of the phenotypic parent-offspring correlation..." be explicit?	We have revised the sentence:<br><br>*Line 466:*<br>When accounting for indirect assortative mating, we found that 62% of the phenotypic parent-offspring correlation could be explained by passive genetic transmission, whereas the remaining was explained by direct (15%) and passive (23%) environmental transmission.

*** I wonder how high the ML parameter estimates are actually correlated in these two models (I think this info can be extracted uing the vcov() function.	We have extracted the variance-covariance matrix for the parameters for the both the full iAM-ACE and iAM-COTS models. These are now reported as correlations (cov2cor) in Supplementary Figure 13 (see below), and as raw output at https://osf.io/dznbk/. We did not observe anything out of the ordinary. We note that the correlations between the parameters will depend heavily on how the model is parameterized (e.g., $\tilde{a} = a$ versus $Var(S) = Var(P)$). For example, a and c are typically highly negatively correlated, something we also find if we use a variance constraint. However, when $\tilde{a} = a$, this correlation is positive in the iAM-ACE model. Because we now parameterize the models by fixing $\tilde{a}$, there are no estimated correlations between, say, $\tilde{a}$ and $\tilde{c}$. To find these, one may apply the models to simulated data using different parameterizations using the provided scripts.<br>*Line 670*<br>Correlations between parameter estimates are presented in **Supplementary Figure 13**.

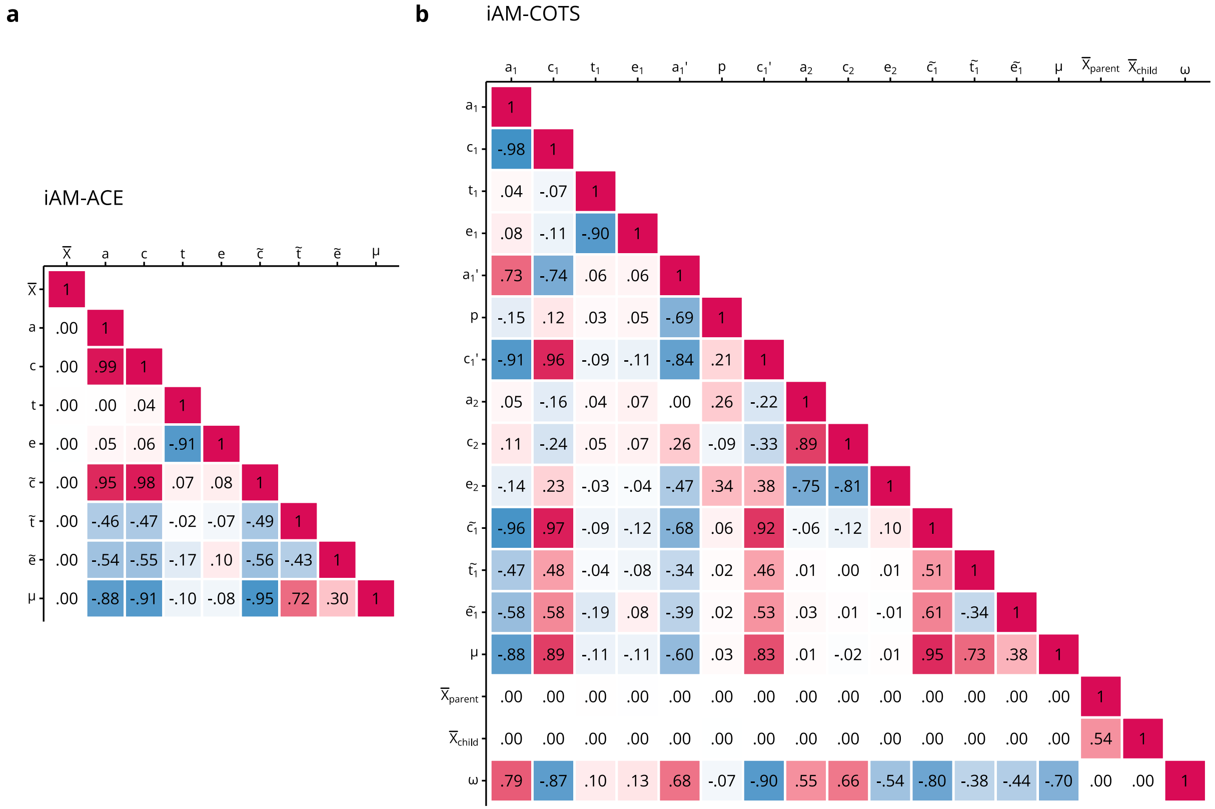

Reviewer #3's comments	Our response and relevant changes
The authors have done a responsible job in responding to reviewers. My main struggle with their work is that is 'confined' to their own framework ('the framework we present') which employs refined definitions of common terminology. For example, AM is not AM when it cause is social stratification. Many researchers will define any resemblance between spouses as AM. There are many more examples of 'under definitions'. NB see response to R3 (line 59): this seems to imply that any spouse resemblance maybe called AM.	While one occasionally sees the term "assortative mating" incorrectly used to refer to any partner resemblance, our impression is that most researchers investigating partner similarity correctly distinguish assortative mating from other causes of partner similarity such as convergence. This distinction is not confined to our framework but is generally accepted in the field. However, we agree that this point can be made more forcefully, and have revised accordingly:<br><br>*Line 58:*<br>A defining feature of assortative mating as opposed to other causes of partner similarity is therefore that it induces covariance without affecting variance.
The motivation for the paper -modified in abstract- is to gain understaning on intergeneration transmission: could the authors add a conclusion after the analysis of the impressive dataset, about what we learned regarding this form of transmission?	We have made a small revision to the opening paragraph in the discussion to highlight what we learned about intergenerational transmission:<br><br>*Line 463:*<br>[...]. Finally, we have shown that attempts at decomposing intergenerational correlations are sensitive to assumptions about type of assortative mating. For traits like educational attainment, the importance of environmental transmission may be understated if failing to account for indirect assortment. When accounting for indirect assortative mating, we found that 62% of the phenotypic parent-offspring correlation could be explained by passive genetic transmission, whereas the remaining was explained by direct (15%) and passive (23%) environmental transmission.
As the second aim of the paper (resposne to R2) is to study EA, this should be reflected in title.	We have now modified the title to reflect that the paper focus on educational attainment:<br><br>*Title:*<br>Understanding indirect assortative mating and its intergenerational consequences for educational attainment

EA is a trait with high spouse correlations, which as not seen for most other traits. How much would a reseracher gain by applying this new framework to other traits?	We appreciate this insightful question. The framework, which provides refined definitions of genetic and social homogamy and how they relate to other causes of partner similarity, is broadly applicable across traits, regardless of the magnitude of partner correlations. Even for traits with lower partner correlations – such as personality and mental health – the framework can help clarify distinctions between different sources of partner similarity, reduce conceptual ambiguity, and facilitate clearer research questions and presentation of results. And partner similarity for traits with lower partner correlations should be studied. For example, even mild assortative mating can have meaningful consequences for genetic analysis, especially when it co-occurs with even mild vertical transmission (see e.g., preprint by Border et al 2024).<br><br>The models we have developed apply this framework to estimate the degree of genetic and social homogamy. While we believe they can be useful for traits where assortative mating is present, we acknowledge that their applicability will be constrained by statistical power and data availability. However, additional models could be developed based on this framework to accommodate different traits, data structures, and research questions.
